# Microneedles as Gateways: Smart Nanoparticle Delivery for Enhanced Breast Cancer Treatment

**DOI:** 10.1021/acsomega.5c04565

**Published:** 2025-09-12

**Authors:** Viola Colaco, Deepanjan Datta, Ritu Kudarha, Abhishek Kumar Singh, Namdev Dhas

**Affiliations:** † Department of Pharmaceutics, Manipal College of Pharmaceutical Sciences, 76793 Manipal Academy of Higher Education, Manipal 576104, Karnataka State, India; ‡ Manipal Centre for Biotherapeutics Research, Manipal Academy of Higher Education, Manipal 576104, Karnataka, India

## Abstract

Breast cancer is a common and potentially fatal disease caused by the abnormal proliferation of cells in the breast tissue. This outlines the critical need for early detection and awareness for effective prevention and treatment. Current therapeutic approaches for breast cancer include surgery, chemotherapy, hormonal therapy, and radiation therapy. Even with the promising strides made in breast cancer research and the inevitable advent of new treatments and drugs, achieving optimal therapeutic outcomes remains challenging due to various obstacles. Treatment with side effects is one of the greatest challenges arising from nonspecificity, with multidrug resistance demanding prolonged doses to ensure patients’ quality of life. Nanomedicine has emerged as a revolutionary approach for improving breast cancer therapy by leveraging nanoparticles for targeted drug delivery, enhanced biocompatibility and reduced systemic toxicity. Despite these advantages, nanoparticle-based therapies face challenges, including limited tumor penetration, off-target toxicity, and clinical translation barrier. To overcome these challenges, microneedle technology has been introduced as a minimally invasive, patient-friendly platform for localized drug delivery. MNs facilitate the direct transdermal administration of therapeutic agents, enhancing drug bioavailability at tumor sites while minimizing systemic side effects. The integration of NPs into MN systems represents a novel strategy to optimize cancer treatment by ensuring controlled and precise drug release. This review explores the role of nanoparticles in breast cancer therapy, the design and fabrication of MNs, and the synergies between these two technologies, while also addressing challenges in clinical translation, regulatory frameworks, and future perspectives in cancer nanomedicine.

## Graphical abstract

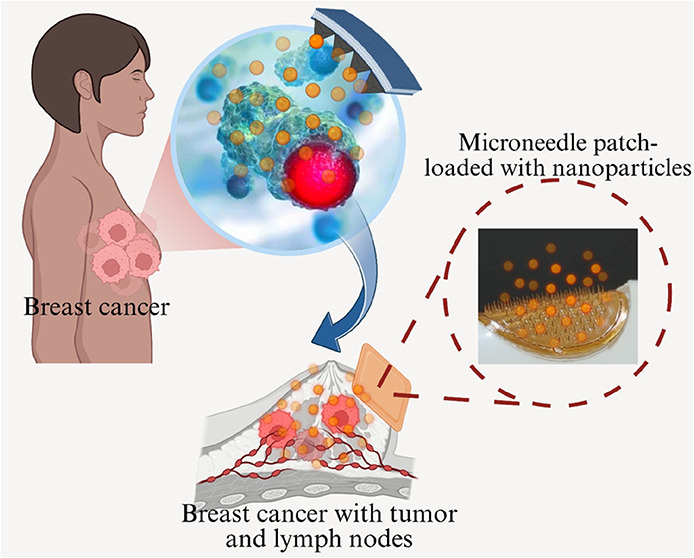

## 1 Introduction

Breast cancer (BC) ranked in second place among diagnosed cancers worldwide in 2022. In 2024, in the United States, BC was estimated to result in 313,510 new cases, including 2790 cases in males and 310,720 cases in females. Similarly, it is associated with an estimated 42,780 deaths, comprising 530 male deaths and 42,250 female deaths (Figure 1).^1^ It is the most common cancer in women and the leading cause of cancer-related mortality in 112 countries.^2,3^ It is divided into three primary types: estrogen receptor-positive (ER+), human epidermal growth factor receptor 2-positive (HER2+) and triple-negative breast cancer (TNBC).^4^ Even with advances in the early detection and treatment of BC, metastasis is still the most common cause of death for patients with this disease, and the 5-year survival rate is less than 30%. The mechanism underlying metastasis is crucial to designing effective therapeutic approaches.^5^ Advances achieved through surgery, radiation, chemotherapy, hormone therapy and targeted therapy greatly enhance patient outcomes.^6^

**Figure 1:**
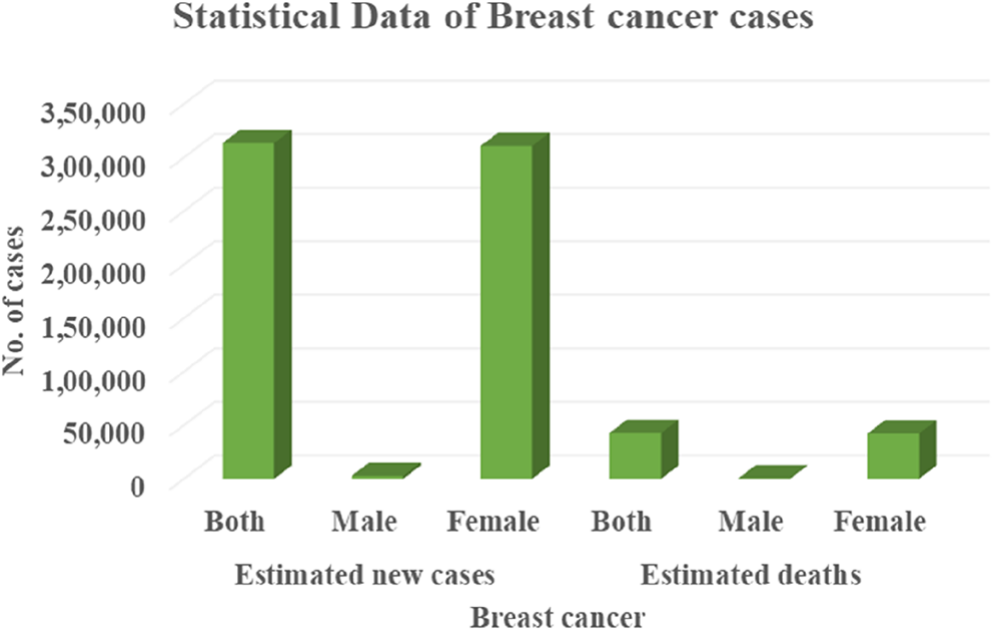
Estimated breast cancer cases and deaths in the United States (2024).

The BC treatment strategies are tailored to the disease stage, molecular subtype and individual patient factors. Early stage management typically involves surgery (lumpectomy or mastectomy), radiation and systemic therapies like chemotherapy or hormonal therapy, with neoadjuvant approaches increasingly used for HER2+ and TNBC. Locally advanced BC is managed with neoadjuvant chemotherapy to reduce tumor burden, followed by surgery, radiation and auxiliary node management.^7,8^ Metastatic BC treatment is mainly systemic, including chemotherapy, targeted agents like trastuzumab, PARP inhibitors and CDK4/6 inhibitors; immunotherapy or hormonal therapy is given with an emphasis on palliative care for the relief of symptoms.^9^ Radiation therapy has been integral in local control by including Whole Breast Radiotherapy (WBRT), Accelerated Partial Breast Irradiation (APBI) and Postmastectomy Radiation Therapy (PMRT), which are tailored by stage and risk, while Intensity-Modulated Radiation Therapy (IMRT) has allowed for reduced toxicity.^10^ Precision and minimally invasive therapeutic approaches have been significantly advanced. However, challenges remain in treatment-related complications, specifically lymphedema, pneumonitis and secondary malignancies, underscoring the need for patient-specific planning and further innovation.

Nanomedicine uses nanoscale materials ranging from 1 to 100 nm in size to develop new therapeutic drugs and medical devices. The nanomaterials exhibit properties such as high surface-to-volume ratio, enhanced conductivity, supermagnetic behavior, optical absorption spectra, and characteristic fluorescence features. These characteristics allow nanomaterials to facilitate drug delivery, increase biocompatibility and transcend biological barriers.^11,12^ Hence, their use in targeted cancer therapy is essential for treatment, especially since they can easily target specific biomolecules to enhance treatment efficacy without toxic effects on normal cells. Nanomedicine has advanced the treatment of BC by countering the limitations of conventional therapies. Systemic anticancer drugs, including anthracyclines and taxanes, which are typically used in chemotherapy in the late stages of BC, suffer from problems like hydrophobicity, poor targeting, and high toxicity.^13,14^ Nanomedicines are revolutionizing this by protecting the drugs from being degraded in the biological environment; they improve the targeting of anticancer drugs toward cancer tissues, as well as improving biocompatibility; nanomedicine reduces the adverse effects, as well as ensures a higher drug concentration at tumor sites. In addition to that, it may target and eliminate BC stem cells, critical in the beginning of cancer, as well as the recurrence and resistance toward chemotherapy and radiotherapy.^15,16^ FDA-approved and investigational nanoparticle (NP)-loaded platforms, such as liposomes and polymeric nanoparticles, have been quite promising in BC therapy.^13^ These formulations bring out the advantages of nanotechnology in improving therapeutic outcomes and reducing systemic toxicity.

Although NP-based delivery systems showed great promise in improving BC therapy, their systemic delivery had challenges like limited delivery into the tumor, off-target toxicity, and patient compliance.^17^ Beyond limitations, microneedle (MN) technology is emerging as a good strategy for drug delivery systems with minimized invasiveness, enabling localized dosage delivery of efficacious therapeutics while minimizing the risk of systemic side effects. For example, MN deploys chemotherapeutic agents within the skin, thus leading to a higher concentration of chemotherapeutic agents at the tumor site and lowering the requirement for high doses that could potentially cause damage to healthy tissues.^18–20^ MNs can bypass the epidermal surface and reach the lymphatic capillaries; therefore, they enhance drug distribution and therapeutic benefits. Moreover, it can achieve that with minimal discomfort to the patient. MN is an ideal platform to incorporate NP-based formulations. The addition of NPs to MNs represents a novel approach toward effective therapy that minimizes side effects and ultimately optimizes patient outcomes for BC management.^21^ This article reviews the invention of MNs containing NPs as a promising strategy for BC therapy that overcomes the drawbacks of current therapies. It begins with an overview of NPs in BC therapy regarding their types, mechanisms of action, and the challenges they face. It goes on to detail the design, fabrication, mechanism of release, benefits, and limitations of MN technologies, focusing specifically on their role in cancer treatment. The rationale and some of the applications of integrating NPs into the MNs are underscored with special relevance to their use as therapeutic vehicles; the problems translating to the clinic in future, biocompatibility, and regulatory challenges were discussed. This review uniquely consolidates current advancements in NP-integrated MN systems for breast cancer therapy, offering a comprehensive perspective on their design, therapeutic mechanisms and translational challenges, making it an innovative contribution to evaluate this emerging interdisciplinary strategy.

The various types of nanoparticles employed for BC therapy, including polymeric nanoparticles, solid lipid nanocarriers, nanostructured lipid carriers, mesoporous silica nanoparticles, and gold nanoparticles, have been studied (Supporting Information, Section S1, Figure S1).

## 2 Targeting Strategies To Improve The Efficiency of NPs in BC

The NPs offer transformative potential in BC therapy by enabling targeted drug delivery, reducing off-target toxicity, and enhancing therapeutic efficacy through the EPR effect. NPs improve drug delivery through biocompatibility, immune evasion, and site-specific targeting, which show promise in theranostics. Nanocarriers functionalized with ligands and polymers like PEG evade immune detection, prolong circulation and offer controlled, stimuli-responsive drug release while bypassing multidrug resistance mechanisms. Advances in multifunctional nanocarriers for combination therapy, prodrug-based nanomedicines for precision treatment.^22,23^ NP-based therapeutic strategies for treating cancer are broadly divided into passive targeting and active targeting (see Supporting Information, Section S2, Figure S2).

## 3 Painless Puncture: The Rise of Microneedle Technology

The skin, the body’s largest organ, serves as a protective barrier against environmental aggressors and pathogens while limiting the passive transport of compounds with suboptimal physicochemical properties.^24^ However, the skin’s barrier limits passive transport to drugs with specific properties, such as molecular weight under 500 Da and log P between 2 and 3, due to the barrier function of the stratum corneum (SC). The transdermal route offers a noninvasive method of drug administration with improved patient compliance, high bioavailability, bypassing gastrointestinal degradation and controlled release. Drug accumulation in the skin before release into the bloodstream provides sustained delivery.^24–27^ The MNs consist of a patch base with micron-scale needles attached, and they have garnered significant attention in research over the past few decades. Although the term “MNs” was introduced in 1998, the concept was initially proposed in the 1970s by Pistor as a transdermal delivery device.^18,28,29^ Typically, MNs feature arrays of micro projections with dimensions ranging from 500 to 900 μm in length, 50 to 250 μm in width and 1 to 25 μm in thickness.^19,30^ MN technologies are fabricated using various materials and fabrication methods tailored to specific applications, including drug delivery, diagnosis and cosmetics. The selection of MN type, material and fabrication technique is closely linked to its intended application.^18^ Drug delivery through the topical route primarily occurs via diffusion. In the MN-based system, the skin barrier is temporarily disrupted using an array of MNs arranged on the patch. These MNs pierce the SC, bypassing the barrier layer and depositing the drug into the epidermis or upper dermis.^31^ Additionally, the MN patch strives to minimize contact with nerve endings and blood vessels, thus decreasing pain during MN applications.^24^ Since their inception, extensive studies have explored their potential for therapeutic, diagnostic and biomedical applications, leading to the commercialization of numerous MN-based technologies in recent years. Detailed information on microneedle types and their release mechanisms is summarized in Supporting Information (Section S3, Figure S3, Table S1), while design parameters and fabrication approaches are described in Supporting Information (Sections S4 and S5).

Among the various MN types, dissolvable and hydrogel-forming MNs are particularly relevant for NP-based drug delivery due to their biodegradability, biocompatibility, and potential for transdermal controlled release. These two systems exhibit distinct release profiles and therapeutic advantages, making them suitable for different clinical contexts. Dissolvable MNs are fabricated from water-soluble polymers that rapidly disintegrate upon skin insertion, releasing the encapsulated payload. This fast-release mechanism is advantageous for vaccines or acute dosing.^32^ Bhatnagar et al. developed DMNs composed of PVA and PVP, enabling rapid and efficient codelivery of doxorubicin (DOX) and docetaxel, with complete needle dissolution within 1 h and near-complete drug release within 15 min. *Ex vivo* permeation studies demonstrated substantial skin penetration, with up to 73% of DOX and 33% of docetaxel delivered across murine skin in 48 h. *In vivo* studies using a 4T1 breast cancer xenograft model showed that DMN-based delivery significantly reduced tumor volume and improved survival rates compared to intratumoral injections, which caused severe toxicity and mortality. The DMNs provided sustained intratumoral drug retention, minimized systemic side effects, and enhanced therapeutic efficacy.^33^ In another study, Patil and colleagues formulated artemisinin (ART), ART-LBL-MN, which demonstrated enhanced transpapillary delivery and release efficiency due to improved solubility, nanosizing, and lipid entrapment. The LBL coating with chitosan and HA enabled targeted CD44-mediated uptake and sustained drug release. *In vitro* and *ex vivo* studies showed rapid initial release with ART-SLN-MN, while ART-LBL-MN exhibited prolonged release due to the coating barrier.^34^

In contrast, hydrogel-forming MNs swell upon exposure to interstitial fluid, forming continuous conduits for sustained or stimuli-responsive drug release. These MNs are ideal for chronic therapies or complex release profiles.^35^ The pH-responsive MPEG-PAE micelles rapidly disassembled under acidic TMEs, enabling a burst release within 4 h and sustained drug release for up to 4 days. The acidic tumor environment triggered micelle disassembly, enhancing drug release and deep tumor penetration. Compared to oral or intravenous routes, MN delivery achieved superior tumor targeting (87.7% localized accumulation) and prolonged ER degradation, which oral delivery failed to sustain.^36^ Both dissolvable and hydrogel-forming MNs demonstrate high potential for NP-mediated BC therapy, with the selection dependent on therapeutic goal. Integrating NPs within these MN platforms provides a customizable approach for enhancing efficacy, safety and patient compliance in BC care.

### 3.1 Smart/Stimuli-Responsive MN Systems

Smart/stimuli-responsive MNs represent a cutting-edge approach for transdermal drug delivery systems engineered to respond to specific internal (e.g., pH, enzyme activity, hypoxia) or external (e.g., light, photo, ultrasound, electric fields) stimuli (Figure 2). These systems enable site-specific, on-demand drug release of therapeutic agents such as chemotherapeutics, photosensitizers, immune modulators, or nucleic acids, enhancing precision and minimizing systemic toxicity. By integrating diagnostics and therapy, they function in a “closed-loop” manner, responding to disease-associated cues to regulate therapeutic outcomes with greater control, efficiency, and safety. Compared to conventional MNs and topical formulations, these systems offer significant advantages such as painlessness, ease of use, minimal invasiveness, and improved patient compliance.^37–39^

**Figure 2:**
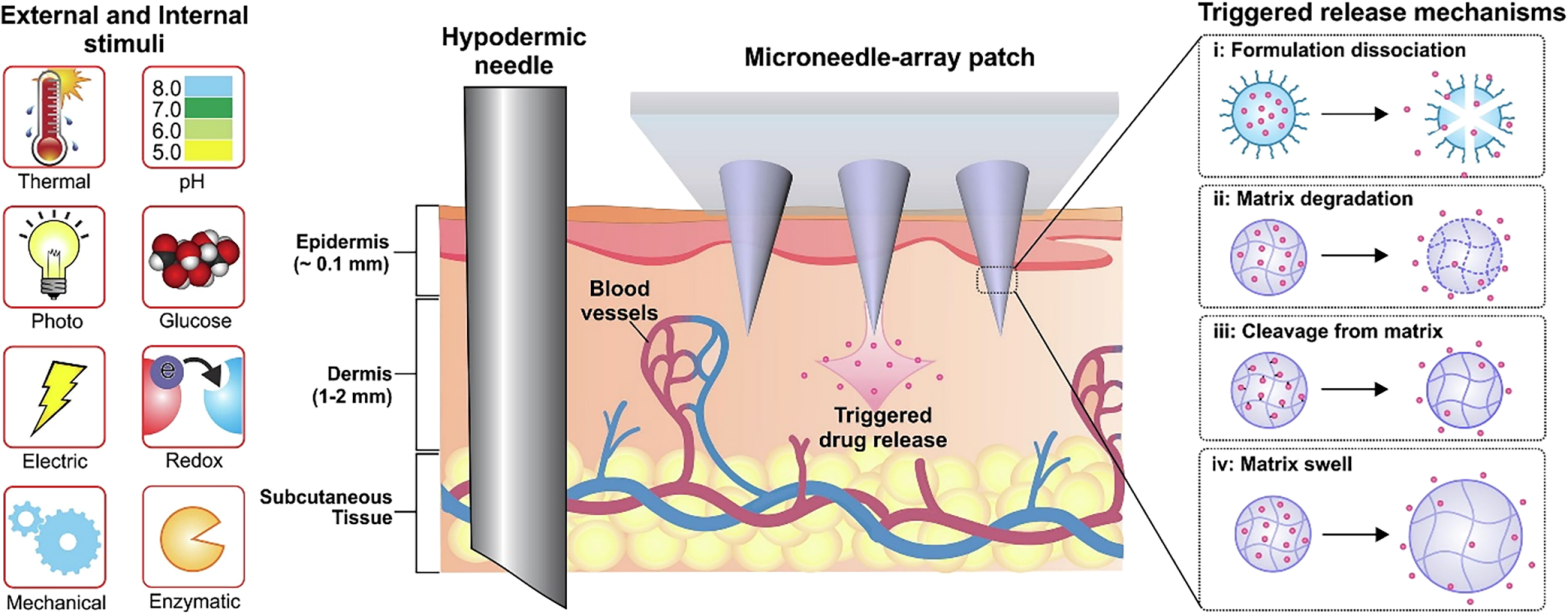
Various types of internal and external stimuli, along with their corresponding trigger-release mechanisms, are involved in the stimulus-responsive transdermal MN patch. Adapted with permission from ref 40. This is an open-access article, available under the terms of the Creative Common CC-BY license. Copyright 2021, Pooyan Makvandi.

Wang et al. fabricated stimuli-responsive separable MN system coloaded with the photothermal NIR-II fluorophore Flav7 and DOX for light-triggered chemo-thermal therapy for superficial tumors. The MNs consist of phase-change PCL arrowheads, which melt under NIR irradiation, enabling on-demand drug release, and PVA/PVP bases that dissolve to deposit the arrowheads into the skin. The MNs exhibited strong mechanical strength for effective skin penetration and repeatable photothermal behavior upon NIR activation. Under light stimulation, Flav7 enabled localized heating, melting the PCL tips and releasing DOX in a controllable, on/off fashion, demonstrating precise spatiotemporal drug delivery. *In vivo* NIR-II fluorescence imaging revealed that Flav7 provided high signal intensity at the tumor site, peaking at 24 h with an SNR of 8.67, and was efficiently cleared within 48 h, indicating excellent imaging performance and low systemic accumulation. In a 4T1 breast tumor model, MN-treated mice showed rapid temperature rise (50–53 °C) under NIR irradiation, sufficient to melt the PCL tip and release DOX. The combination group (Flav7+DOX+NIR) exhibited severe tumor cell necrosis, suppressed proliferation, and complete tumor elimination, with a 100% survival rate over 50 days and no significant body weight loss. H&E staining of major organs confirmed no detectable toxicity, supporting the biosafety of this stimuli-responsive theranostic MN platform for precise breast cancer treatment (see Supporting Information, Figure S5).^41^

An MN patch using vinylpyrrolidone-vinyl acetate copolymer (PVPVA) to codeliver photosensitizer IR820 and cisplatin (CIS) for synergistic chemo-photodynamic therapy against BC was fabricated by Fu et al. The MN patch was fabricated via a two-step centrifugation casting method and demonstrated high encapsulation efficiency (89%) and excellent mechanical stability. Upon NIR radiation, IR820 produced ROS, enhancing the photodynamic effect without decreasing CIS chemotherapeutic efficacy. The *in vitro* studies showed synergistic cytotoxicity in 4T1 cells, with increased apoptosis and reduced cell viability compared to monotherapies. *In vivo* studies, the MN patches effectively suppressed tumor growth in 4T1 tumor-bearing mice, achieving a tumor inhibition ratio of 90% with minimal systemic toxicity or organ damage. The tumor inhibition in the single-drug MN groups was moderate (52 and 64% for CIS and IR820, respectively). Histological analysis showed the highest apoptosis and lowest proliferation rates in tumors treated with CIS-IR820-MN patches, as evidenced by TUNEL and cleaved-caspase-3 staining.^42^

In another study, Cheng and colleagues developed a pH-responsive MN patch for localized delivery of PROTACs (ERD308) and palbociclib, a CDK4/6 inhibitor to treat ER-positive BC. The drugs were coencapsulated in MPEG-PAE micelles, a pH-sensitive polymer that rapidly demicellizes in the acidic TME, enabling targeted and sustained drug release. These micelles were loaded into cross-linked hyaluronic acid–based MNs, which exhibited strong mechanical strength to penetrate breast tissue. These micelles remain stable at physiological pH (7.4) but rapidly dissociate under mildly acidic conditions (pH ∼ 6.4), commonly found in tumor tissues. *In vitro*, the micelles showed pH-triggered drug release, enhanced cellular uptake (18.5-fold increase), and potent ER degradation and cytotoxicity against MCF7 cells, with minimal toxicity to normal cells. *In vivo* studies, MN patch demonstrated superior tumor-targeted biodistribution (87.7% localized in tumors), deep tumor penetration, and sustained ER degradation for up to 4 days—surpassing oral PROTAC delivery. this system represents a smart transdermal platform for responsive, sustained and localized cancer therapy.^43^

A stimuli-responsive MN platform for enhanced PDT in hypoxic tumors was reported by Liu et al. The system comprises chlorin e6 (Ce6)-loaded DMN embedded with sodium percarbonate (SPC) as an oxygen propellant. Upon insertion into the skin, the polyvinylpyrrolidone-based MNs dissolve, allowing SPC to react with interstitial fluid and generate gaseous oxygen. This moisture-triggered oxygen release produces convective flows that actively enhance Ce6 penetration and alleviate tumor hypoxia. SPC-loaded DMNs generated oxygen upon insertion, enhancing Ce6 penetration (1130 ± 65 μm vs 918 ± 12 μm with SC controls) in hypoxic 4T1 cells, ROS levels increased 2.37-fold under laser irradiation, and Ce6-SPC DMNs reduced cell viability to 1%, confirming enhanced phototoxicity. Agarose gel simulations further validated superior ROS production and cytotoxicity due to oxygen-enhanced delivery. SPC-DMNs showed adequate mechanical strength for skin insertion, with full recovery and no inflammation within 24 h. In 4T1 tumor-bearing mice, Ce6-SPC DMNs combined with laser significantly inhibited tumor growth and improved survival. Elevated intratumoral ROS and hypoxia relief supported the enhanced PDT efficacy. No systemic toxicity or organ damage was observed, confirming biocompatibility and treatment safety.^44^

Zandi and colleagues developed silicon-based MNs integrated with electrodes and decorated with vertically aligned ZnO nanowires (ZnONWs) to electrochemically generate microbubbles (MBs) *in vivo* by electrolyzing interstitial fluid under pulsed DC voltage (−2 V, 1 ms). Upon ultrasound stimulation, these MBs induced localized cavitation, facilitating cell membrane perforation (sonoporation) and enhanced intratumoral uptake of paclitaxel (PTX). In MDA-MB-231 cells, the combination of PTX, ultrasound (US), and MBs (G5 group) reduced cell viability by ∼52%, significantly greater than PTX alone (G3, ∼11%) or PTX+US (G4, ∼20%). FESEM and confocal imaging confirmed membrane sonopores facilitating drug uptake. ZnONWs showed no cytotoxicity or cell cycle disruption, confirming *in vitro* biocompatibility. In BALB/c mice, ZnONW-MGPs produced localized MBs (lifespan 6–11 s) upon activation, confirmed via sonography. Histology (H&E), IHC (P53), and serum markers (AST, ALT, urea, creatinine) confirmed no systemic toxicity. In an MC4L2 breast cancer model, G5 (PTX+MBs+US) achieved 82% tumor reduction, superior to G3 (3% increase) and G4 (15% reduction). G5 tumors showed elevated P53, reduced Ki67, and widespread apoptosis. PET and X-ray imaging revealed no metastasis, and Kaplan–Meier survival curves demonstrated complete tumor remission and highest survival in G5.^45^

Stimuli-responsive MN systems represent a significant advancement in precision drug delivery, offering spatiotemporal control, minimal invasiveness, and a smart responsive system. Their ability to respond to disease-specific triggers ensures effective therapy while minimizing risks of under- or overmedication. Despite encouraging preclinical outcomes, several hurdles must be addressed before clinical translation: variability in skin penetration, preservation of bioactive components during fabrication, scale-up challenges, and incomplete clinical safety validation. Moreover, establishing regulatory standards for medical MNs remains crucial. Nonetheless, the continuous evolution of smart MN technologies and growing *in vivo* evidence underscore their significant potential for future clinical applications in chronic disease management and beyond.

## 4 Smart Delivery of MNs-Loaded NPs for Targeted Breast Cancer Therapy

MNs were developed in the 1970s. It is a noninvasive and innovative alternative to traditional drug delivery systems. Being fabricated as micron-sized needles arranged in the patch, MNs enable the localized, controlled, and painless transdermal delivery of therapeutic agents such as drugs, vaccines, and genes directly to tumor sites (see Supporting Information, Table S2). It indeed enhances safety, supports self-administration, and reduces systemic side effects, making MNs a promising platform for cancer treatment. Traditional parenteral drug delivery methods are often hindered by poor targeting, rapid clearance, and toxicity to nontarget organs. Similarly, oral and buccal routes face challenges such as enzymatic degradation in the gastrointestinal tract, limiting therapeutic efficacy. These limitations underscore the need for advanced delivery platforms like MNs.^46^

Cancer therapy is a multifaceted process, typically commencing with diagnosis and followed by treatment strategies tailored to various factors such as cancer type, stages, patient health, treatment availability and affordability.^47^ Cancer treatment typically includes chemotherapy, surgery, radiation, and targeted therapies. However, monotherapy falls short in the complete eradication of tumors, prevention of recurrence, or prolonging the lifespan of a patient, calling for a multimodal approach combining systemic therapies, including preoperative, postoperative, and both treatments.^18^ MNs offer an innovative way to tackle these challenges through localized drug delivery. By delivering therapeutics directly to the tumor site, MNs limit systemic exposure and increase drug bioavailability. Thus, precise delivery minimizes the need for higher or frequent doses and the overall burden on patients. Moreover, MN systems are minimally invasive and allow these cancer patients to go through various rounds of treatment with greater comfort and compliance. Therefore, newer technologies such as MNs offer encouraging solutions in overcoming challenges related to such treatment because of reduced side effects and pain.^18^ Recent studies have highlighted the advantages of employing MN patches and combination therapies where the synergism of drugs or treatment modalities enhances therapeutic efficacy. These systems guarantee therapeutic outcomes with a high level of reproducibility and accuracy, which is a further assurance of their role in future cancer treatment.^48,49^ Moreover, MNs have shown great promise in the diagnosis of cancer. These reach the interstitial fluid (ISF) in dermal and subcutaneous tissues.^50^ It is well established that ISF contains specific required biomarkers for diagnostics, metabolites, and cancer detection of minimal invasiveness. Recent evidence has revealed that ISF diagnostics can tell when BC biomarkers appear as much as 7 days earlier than serum analysis, thus providing greater insight into early tumor development.^51,52^

The NP formulations are increasingly explored for delivering therapeutic agents, as they offer several advantages, including size-dependent physicochemical properties, protection of payload from degradation, controlled and prolonged drug release and targeted delivery to specific sites and reducing adverse effects.^21^ The integration of NPs into MN systems for transdermal drug delivery necessitates careful consideration of their physicochemical properties to ensure optimal therapeutic performance. Traditionally, NPs in the size range of 10–200 nm have been favored due to their enhanced skin permeability, via follicular or intracellular routes and their ability to facilitate cellular uptake and systemic delivery when combined with MNs.^53^ However, recent advancements have demonstrated that larger particles ranging from 300 nm to 3.5 μm can also be effectively incorporated into MN systems without compromising mechanical integrity or insertion efficiency.^54^ Additionally, zeta potential is a critical parameter of the nanoparticle that influences its behavior during MN-mediated delivery. Studies have shown that NPs with moderate negative surface charge (−30 to −40 mV) maintain colloidal stability, minimize aggregation, and effectively permeate the microchannels created by MNs.^55^ Transdermal drug delivery (TDD) is a promising route that minimizes complications linked with oral and IV administration. NPs can penetrate the SC barrier without disrupting it by leveraging their small size and large surface area, employing transcellular or transappendageal pathways.^56^ However, SC still represents a significant barrier to the efficient translocation of NPs.

Future advancements and hybrids of current technologies are required to address these challenges and improve the performance of NP-based skin permeation drugs.^21^ Compared to small-molecule drugs, drug-laden NPs have shown better capabilities in passing through the micropores created by MNs and hold advantages over micron-scale particles in overcoming the skin barrier. Historically, MN technology has established itself as a versatile platform for delivering NPs in a minimally invasive manner through the skin. The combination of NPs with MN arrays provides a revolutionary mode of drug delivery. This approach addresses problems like hydrophobicity and low permeability, leading to the creation of a multifaceted pharmaceutical platform capable of excellently delivering local therapeutic agents for disease treatments.^57^ In recent years, intradermal NP delivery via MNs has gained much attention due to their unique abilities to facilitate transdermal administration, deliver both hydrophilic and lipophilic therapeutic agents, enable a homogeneous distribution of NP-based drug reservoirs within the skin, and integrate therapeutic and diagnostic functionalities into a single theranostic system.^21,56,58^ Research showed that combining NPs with MNs synergistically enhances skin permeation, due to the reversible disruption of the skin barriers by MNs and subsequent delivery of NPs (see Supporting Information, Figure S6).

The NP integration with MNs has enabled accurate and effective delivery of antitumor agents, chemotherapeutics, and gene therapies.^59^ Current studies have indicated the promise of MNs containing NPs in targeting chemotherapy drugs such as DOX and cisplatin through improved lymphatic targeting and tumor suppression.^59–61^ This new platform builds on the beneficial aspects of NPs and MNs to tackle several limitations of conventional cancer therapies. This provides a minimally invasive approach toward tumor management and immunotherapy. An implantable MN system was developed by Zhang et al., for targeted treatment of TNBC by delivering engineered *Escherichia coli (E. coli)* conjugated with gold nanorods@cerium–zinc composite core–shell nanoparticles (Au@Zn/CeO) (AZCE). The MNs were formulated using PVP, HA, glucose and glycerol to enable localized delivery. upon implantation, AZCE penetrated the ECM and basement membrane, enhancing NP delivery to cancer stem cell (CSC)-rich tumor regions. AZCE released Zn^2+^ and Ce^3+^/Ce^4+^ in the TME, inducing mitochondrial damage, depleting glutathione, elevating reactive oxygen species (ROS), and disrupting redox homeostasis. Under NIR irradiation, the Au nanorods facilitated photothermal therapy (PTT), further enhancing therapeutic effects. The *in vitro* studies in 4T1 cells demonstrated superior CSC spheroid penetration and therapeutic efficacy of AZCE-MN, especially under NIR exposure. Further, *in vivo* studies conducted on female BALB/c mice showed that AZCE-MN in the presence of NIR significantly inhibited tumor growth and CSC proliferation without systemic toxicity. This confirms enhanced cellular uptake, ROS production, CSC apoptosis, and tumor inhibition (see Supporting Information, Figure S7).^62^

Jha et al. presented a dissolving MN drug delivery system with HA-OA/CS-OA NPTs for the targeted delivery of cabazitaxel (CBT). The rats’ skins were engineered via a polymeric blend (HA, PVA and PVP), wherein the microchannels were created and localized for HA-OA/CS-OA NPT delivery. The hybrid NPs were synthesized using ionic gelation of HA-OA and CS-OA, stabilized through TPGS-COOH, with size optimization toward 112 nm, zeta potential of −10 mV, and high drug load for CBT. The drug release studies revealed pH-responsive release, with higher release at acidic pH (96.22% at 72h). The *in vitro* cellular studies were carried out on MDA-MB-231 cells, where cellular uptake assays demonstrated enhanced internalization via receptor-mediated endocytosis due to CBT. Cytotoxicity studies showed that NPT formulations significantly reduced the IC_50_ value in MDA-MB-231 cells compared to free CBT. Mechanistic studies confirmed mitochondrial membrane potential disruption, G2/M cell cycle arrest, and apoptosis induction. The fabricated MNs (∼546 μm height) demonstrated efficient dissolution, facilitating controlled NP delivery. The *in vivo* studies were carried out on DMBA-induced tumor-bearing female SD rats, which demonstrated that HA-OA/CS-OA NPT-MN significantly inhibited tumor growth, achieving a 27.5-fold and 68-fold reduction in tumor volume compared to CBT-MN and CBT (i.v.), respectively, over 14 days. This superior efficacy was attributed to enhanced tumor site permeation, regioselective delivery, and receptor-mediated intracellular accumulation. Body weight analysis revealed no significant changes in HA-OA/CS-OA NPT-MN-treated groups compared to CBT (i.v.), which induced systemic toxicity. The survival rates were highest in HA-OA/CS-OA NPT-MN-treated groups, with a median survival of more than 120 days, compared to 41 days in untreated rats. Histological analysis confirmed the disappearance of cancer cells and necrotic regions, further validating the therapeutic potential of HA-OA/CS-OA NPT-MN.^63^

In another study by Sharma and colleagues, they fabricated an organic solvent-free MN array composed of HA, PVA and PVP for targeted ribociclib (RB) delivery via HA-functionalized ultradeformable transferosomes (HA-RB-Ts). The formulation demonstrated effective skin penetration with a depth of approximately 341 μm and maintained structural integrity during storage. *In vitro* cell line studies using CD44-overexpressing BC cells (MDA-MB-231 and LA-7) revealed significantly enhanced cytotoxicity and apoptosis for HA-RB-Ts compared to free RB, attributed to receptor-mediated uptake and increased intracellular ROS production. *Ex vivo* and confocal imaging confirmed precise skin insertion and dissolution of the MNs, enabling sustained and targeted drug release. *In vivo* tumor inhibition studies on SD female rats developed an LA-7 syngeneic mammary tumor model, which showed superior efficacy of HA-RB-Ts-MNs, with a 6-fold higher tumor-site drug concentration and a 96% increase in survival rates compared to free RB. Pharmacokinetics studies demonstrated prolonged systemic circulation and reduced accumulation of RB in vital organs, minimizing hepatobiliary and cardiotoxicity. The histopathological analyses further supported the safety and efficacy of HA-RB-Ts-MNs, showing significant tumor apoptosis with negligible off-target toxicity. This transdermal system offers a potent, safe and targeted approach for BC therapy (see Supporting Information, Figure S8).^64^

A chitosan-based MN patch loaded with FA-conjugated silver NPs (FA-AgNPs) and letrozole (LTZ) was fabricated by Batool et al. The MNPs demonstrated efficient fabrication, mechanical strength (1.41 N), high drug entrapment and efficient skin penetration, reaching a depth of 560 μm without irritation. *In vitro* studies showed sustained drug release (45% over 48 h) with a release mechanism aligned with the Korsmeyer-Peppas model. *Ex vivo* permeation studies revealed a superior drug transport (84%) compared to ointment (28%), and MNPs-treated tissues showed higher drug retention (12%). The *in vitro* cell line studies were conducted on MCF-7 cell lines. Cytotoxicity assays demonstrated that MNPs were more effective (IC_50_ of 50 μM) than LTZ alone (IC_50_ of 70 μM) in reducing MCF-7 cell viability, supported by fluorescence imaging and uptake studies. Cellular uptake studies confirmed 3.2-fold higher internalization of FA-AgNPs-LTZ in MCF-7 cells, highlighting the specificity of FA targeting. These findings underscore the potential of FA-AgNP-LTZ-loaded MNPs for safe, effective and targeted BC therapy with enhanced therapeutic efficacy and reduced systemic toxicity.^65^

Wang et al. developed a multifunctional delivery system to enhance breast cancer therapy by integrating CRISPR/Cas9-mediated FBXO44 gene knockout with PTT and PDT. Hydrophobic IR780 and plasmid pFBXO44 were coloaded into redox-responsive cationic nanomicelles (PL: poly­(ethylenimine) and lipoic acid), forming IR780-PL/pFBXO44 NPs, which were further embedded in steerable DMNs. *In vitro* studies conducted on 4T1 cells demonstrated efficient clathrin-mediated uptake and lysosomal escape of IR780-PL/pFBXO44 NPs, upon NIR at 808 nm irradiation, IR780 triggered ROS generation and photochemical internalization (PCI)-enhanced lysosomal escape, facilitating cytoplasmic plasmid release via disulfide bond reduction. The NPs demonstrated strong photothermal effects, high gene transfection efficiency, and excellent tumor cell inhibition *in vitro*. MNs exhibited sufficient mechanical strength and flexibility and maintained NP integrity upon dissolution. *In vivo* studies in female BALB/c mice showed that pretreatment with collagenase MNs effectively degraded tumor extracellular matrix, facilitating deeper and more uniform nanoparticle distribution. Upon 808 nm NIR irradiation, the system enabled controlled photothermal and photodynamic effects alongside CRISPR/Cas9-mediated FBXO44 knockout, collectively enhancing tumor suppression and inhibiting invasion and metastasis (see Supporting Information, Figure S9).^66^

Huang et al. fabricated dissolving MNs, αNP-RNP@DMNs, for codelivery of the Toll-like receptor 7/8 agonist R848 and the immune checkpoint inhibitor aPD-1. Liposomes loaded with R848 (RNPs) and chitosan nanogels (αNPs) encapsulating aPD-1 were optimized, achieving 72 and 57% encapsulation efficiencies, respectively. These nanodrugs demonstrated stable release profiles and synergistic effects when incorporated into DMNs, which were fabricated using a multilayered micromolding process. The DMNs showed efficient skin penetration, drug deposition and transdermal delivery without causing tissue damage. The αNP-RNP@DMNs effectively enhanced DC maturation within tumors and lymph nodes, reversing the immunosuppressive microenvironment in TNBC. *In vitro* studies showed efficient nanodrug uptake by DC2.4 cells via DMN delivery, significantly increasing DC maturation (71%) and cytokine production compared to controls. *In vivo* studies carried out in BALB/c female mice confirmed the superior transdermal delivery and prolonged retention of nanodrugs with DMN administration compared to subcutaneous injection. Biodistribution studies showed sustained fluorescence signals at the administration site and lymph nodes for up to 48 h, with the strongest lymph node fluorescence observed at 36 h. This prolonged retention and enhanced migration of nanodrugs to lymph nodes facilitated DC maturation at this site. The αNP-RNP@DMNs-treated mice exhibited increased DC maturation in the right inguinal lymph nodes compared to controls, significantly outperforming other treatment groups, including intratumoral injection of nanodrug solutions. These results indicated that the DMN system effectively codelivers immune adjuvants and checkpoint inhibitors, synergistically enhancing DC activation and promoting robust antitumor immunity (Figure 3).^67^

**Figure 3:**
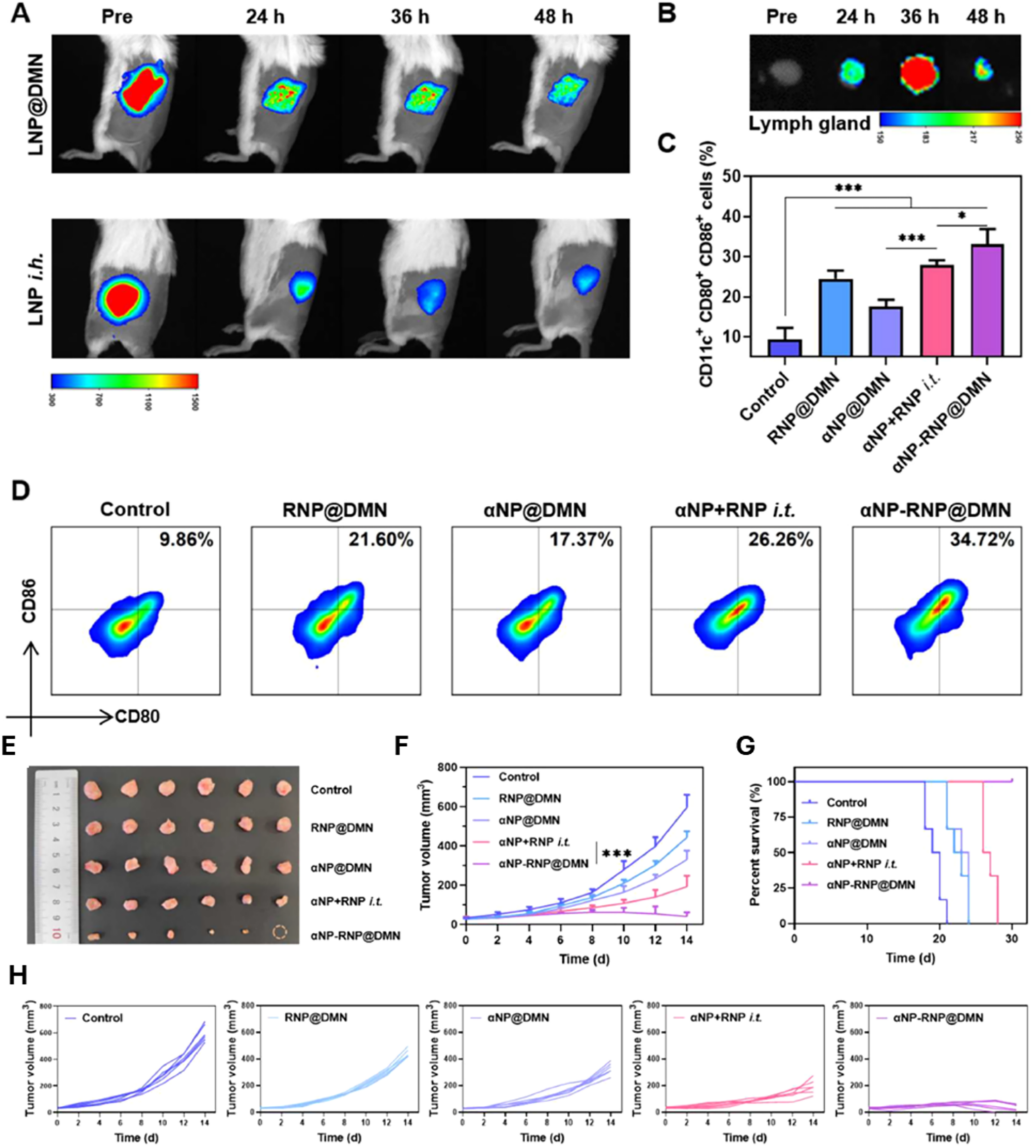
(A) *In vivo* fluorescence images of healthy mice at various time points after inserting DiR-loaded LNP@DMN or subcutaneous injection of DiR-loaded liposomes (LNP i.h.). (B) *Ex vivo* fluorescence imaging of inguinal lymph nodes from healthy mice sacrificed at different time points following treatment with DiR-loaded LNP@DMN. (C, D) Flow cytometry analysis or quantitative analysis of CD80+ CD86+ DCs over CD11c+ DCs within lymph nodes of 4T1 tumor-bearing mice 10 d post-treatment (mean ± SD, *n* = 5, *P < 0.05, ***P < 0.001). *In vivo* antitumor efficacy of αNP-RNP@DMN in 4T1 tumor-bearing mice, (E) Tumor photographs 14 days post-treatment, (F) Tumor volume trends (mean ± SD; *n* = 6; ***P < 0.001). (G) Cumulative survival rates (*n* = 6), and (H) Individual tumor growth curves across treatments. Adapted with permission from ref 67. Copyright 2024, Sicong Huang.

In conclusion, the MNs containing NPs systems have significantly enhanced tumor inhibition, lymphatic targeting, and precise delivery through pH-responsive receptor-mediated mechanisms. Recent studies highlight the synergistic effects of chemo-photothermal therapy, immunotherapy and diagnosis. Innovation in dissolvable MNs, hybrid NP formulations, and theranostics has shown enhanced therapeutic efficacy, prolonged drug release, and reduced off-target effects. These technologies represent a promising future for addressing BC challenges, such as overcoming SC barriers and enabling localized, controlled delivery with reduced side effects.

## 5 Pharmacokinetic and Biodistribution Studies of MNs-Loaded NPs

Pharmacokinetic and biodistribution studies have been conducted in various preclinical models to evaluate the effectiveness of MN-mediated NPs in breast cancer therapy. These studies provide crucial insights into the drug release profiles, systemic circulation behavior, and tissue-specific accumulation patterns of NP formulations administered via MNs.

Resveratrol (RVT)-loaded NLCs were formulated and integrated with a hollow MN array to enhance localized drug delivery by Gadag et al. The NLCs were optimized via statistical modeling for particle size, PDI, zeta potential, and entrapment efficiency, using glyceryl monostearate (GMS), capryol 90, and poloxamer 188 as lipid and surfactant components. *In vitro* studies in MDA-MB-231 breast cancer cells demonstrated superior cytotoxicity, cellular uptake, and migration inhibition by RVT-NLCs compared to free RVT. Incorporation into Carbopol gel allowed controlled release over 48 h, and MN-mediated delivery significantly enhanced skin permeation, with the 1200 MN array showing the highest flux. *In vivo* pharmacokinetic and biodistribution studies in SD rats showed that MN-assisted RVT-NLCs delivery increased systemic bioavailability and sustained plasma levels compared to oral and subcutaneous administration. Importantly, MN application enabled site-specific delivery to breast tissue, achieving higher RVT concentrations and retention than plasma, confirming localized therapeutic potential (Figure 4I). Accelerated stability studies supported the formulation’s stability, and the design space analysis validated the robustness of the system. Overall, the MN-assisted NLC platform presents a promising strategy for localized and sustained breast cancer therapy.^68^

**Figure 4:**
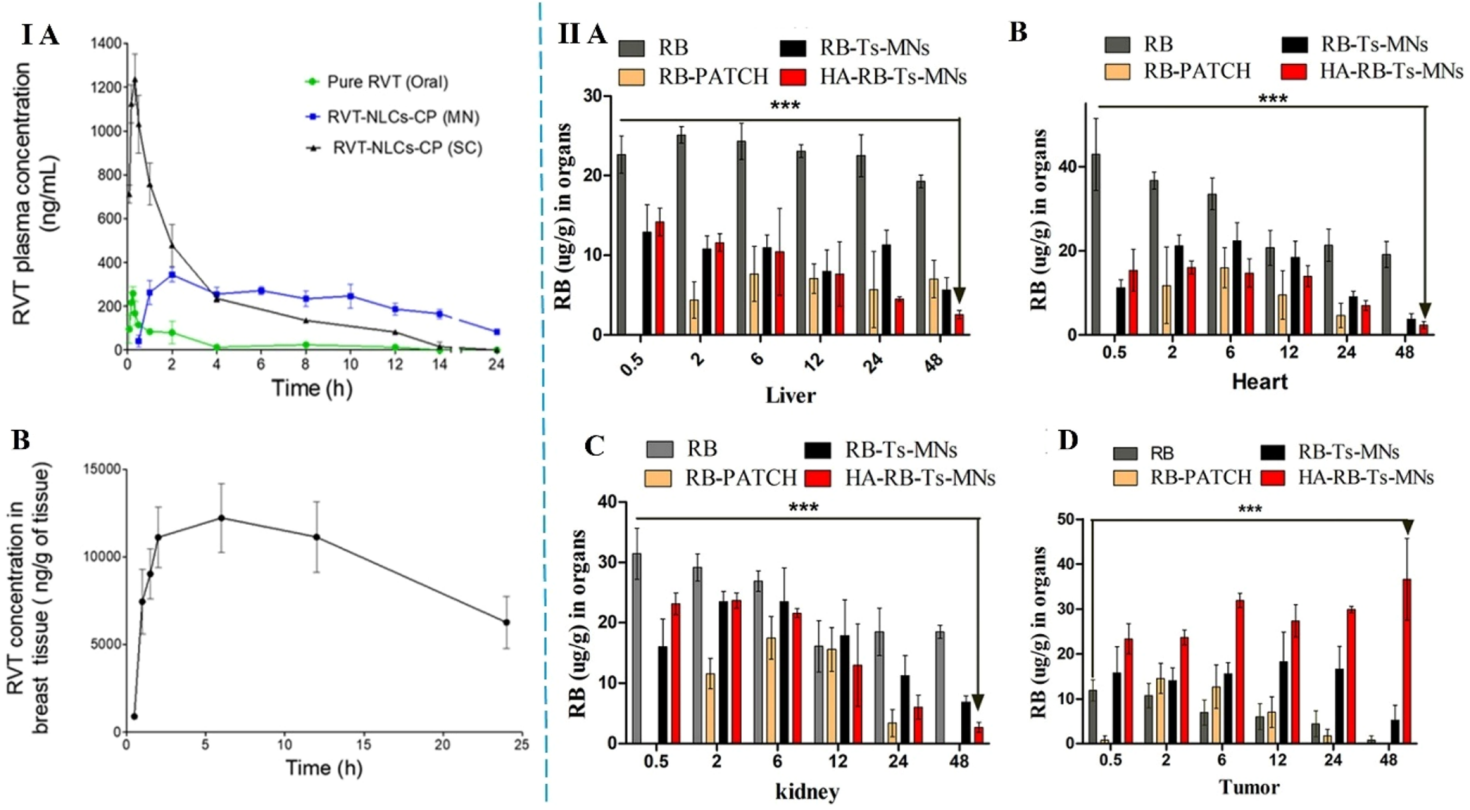
I (A) Plasma concentration–time profile following oral administration of pure RVT, subcutaneous administration of RVT-NLCs-CP, and MN-assisted transdermal delivery of RVT-NLCs-CP, (B) Concentration–time profile of RVT in breast tissue after MN-assisted RVT-NLCs-CP delivery. Adapted with permission from ref 68. Copyright 2021, Shivaprasad Gadag. II post-transdermal application, the biodistribution of RB, RB patch, RB-Ts-MNs, and HA-RB-Ts-MNs was assessed at various time intervals in (A) liver, (B) heart, (C) kidney, and (D) tumor. (****p < 0.001 represents control vs HA-RB-Ts-MNs). Adapted with permission from ref 64. Copyright 2022, American Chemical Society.

The *in vivo* efficacy, pharmacokinetics and biodistribution of HA-conjugated transferosome-loaded MNs (HA-RB-Ts-MNs) were evaluated using a syngeneic LA-7 mammary tumor model in female SD rats by Sharma et al. Tumor-bearing animals were treated via oral or transdermal routes, and comparative analyses revealed that HA-RB-Ts-MNs significantly inhibited tumor growth, with tumor volume and weight reductions 22- and 13.5-fold greater, respectively, than those seen with the free drug. Pharmacokinetic analysis demonstrated a sustained release profile, with HA-RB-Ts-MNs exhibiting a 5.93–8.67-fold higher plasma AUC and prolonged mean retention time compared to other formulations. Biodistribution studies confirmed preferential tumor accumulation with reduced off-target deposition in the heart, kidney, and liver, indicating minimized systemic toxicity. The enhanced therapeutic efficacy was attributed to receptor-mediated active targeting, improved drug penetration, and spatial–temporal release (Figure 4II). Histopathological evaluation further supported reduced hepatotoxicity and cardiotoxicity in MN-treated groups, which was consistent with the biodistribution findings. Additionally, HA-RB-Ts-MNs extended the median survival time of tumor-bearing rats to >45 days with no significant weight loss, highlighting the safety and potential of MN-mediated localized delivery in breast cancer therapy.^64^

Steerable DMNs composed of biocompatible oligo-HA were developed to conform to irregular superficial tumors by Wang et al. IR780-PL/pFBXO44 nanoparticles were incorporated into these DMNs, and pretreatment with collagenase-loaded MNs was employed to degrade the tumor matrix, thereby enhancing deep tumor penetration of the nanoparticles. *In vivo*, enhanced tumor penetration and biodistribution of Col@MNs+IR780-PL/pFBXO44@MNs was observed in 4T1 tumor-bearing mice, as checked through *in vivo* fluorescence imaging. Upon topical administration of Col@MNs+IR780-PL/pFBXO44@MNs, fluorescence signal from IR780 was found to intensively accumulate within the tumor tissue, reaching a maximum at 4 h postapplication. Minimal fluorescence was detected in major off-target organs, indicating low systemic distribution and favorable tumor selectivity. To further validate organ-specific distribution, IR780-loaded nanoparticles were labeled with Cy5.5 dye. *Ex vivo* imaging confirmed predominant accumulation in tumor tissue, with only trace levels in the liver, supporting minimal hepatic uptake and reduced risk of off-target toxicity.^66^

Wang et al. developed a DMNs (PTC NVs@MNs) for transdermal delivery of tumor-derived RNA (TdRNA) nanovaccine composed of protamine (PRT), TdRNA and CpG oligodeoxynucleotide (CpG). The nanovaccine was efficiently encapsulated at the MN tips using a multistep centrifugation method with HA as the matrix. Upon application, dermal dendritic cells (DCs) readily internalized PTC NVs, triggering DC maturation, cytokine secretion, and potent cytotoxic T lymphocyte (CTL) responses, due to the synergistic effect of TdRNA encoding broad-spectrum tumor antigens and CpG acting as an immunostimulatory adjuvant. *In vivo* studies demonstrated the superior antitumor efficacy of PTC NVs@MNs in a subcutaneous 4T1 breast tumor mouse model. Mice treated with PTC NVs@MNs exhibited significantly reduced tumor growth and enhanced survival compared to controls and other delivery formulations, which was attributed to sustained antigen release and prolonged immune stimulation. The pharmacokinetic and biodistribution studies of PTC NVs@MNs revealed prolonged retention at the application site and strong fluorescence signals up to 48 h postadministration, indicating sustained antigen presence. Microneedle delivery enhanced nanovaccine trafficking to draining lymph nodes, promoting dendritic cell activation while minimizing off-target organ distribution.^69^

The pharmacokinetic and biodistribution data strongly support the efficacy of MN-mediated NP delivery in enhancing localized drug accumulation and sustained release in breast tissues. The MN-assisted delivery systems demonstrated prolonged drug retention at the site of application, minimized systemic exposure, and efficient trafficking to tumor or immune-inductive sites like lymph nodes. Notably, RVT-NLC-MNs resulted in superior breast tissue localization with significantly higher AUC and *C*_max_ compared to oral or subcutaneous administration. Similarly, Sharma et al. reported sustained systemic exposure and tumor-specific accumulation with HA-RB-Ts-MNs. At the same time, Wang et al. highlighted enhanced tumor penetration and lymphatic migration for immunotherapeutic agents. Therefore, MNs provide a minimally invasive strategy for targeted, sustained, and safe nanoparticle delivery to breast tumors, enhancing therapeutic efficacy while minimizing systemic toxicity.

## 6 Theranostic MNs for Combined Treatment and Diagnostics of MNs-Loaded NPs

MNs serve as advanced theranostic platforms by combining diagnostic and therapeutic functions. These minimally invasive devices enable real-time sensing of the physiological markers while simultaneously delivering therapeutic agents to the targeted site. This dual functionality supports personalized medicine by allowing disease monitoring, site-specific drug delivery, and prognosis assessment.^70,71^ MN-based theranostic systems offer a promising approach for integrated, patient-specific diagnostics and treatment.

Chen et al. presented a study for early diagnostic method for breast cancer using MNs and breathable MBL-film to collect tissue fluid for a blood-free colorimetric assay based on Ag_3_PO_4_/Ag nanocomposites. The technique enables semilocalized, semiquantitative detection of tumors as early as 7 days post-tumor cell inoculation—significantly earlier than traditional blood tests, which required 14 days. The assay detects carcinoembryonic antigen (CEA) via a pH-dependent colorimetric response using TMB as a chromogenic substrate, with a detection limit as low as 0.2 ng/mL. Animal studies were carried out on nonfertile female SD rats, which showed that early detection via tissue fluid improved therapeutic outcomes with DOX, leading to tumor suppression and increased survival. Human volunteer testing further validated the method’s diagnostic reliability, safety, and sensitivity, demonstrating its potential for early, painless breast cancer screening and monitoring in clinical settings.^72^

In another study, Chen and colleagues developed a multifunctional theranostic MN platform for the precise and immune-activating treatment of TNBC. A porous methacryloyl GelMA-based dissolvable MN patch (MEM) embedded with exosome-encapsulated STING agonist MSA-2 (EM) was fabricated to facilitate localized and sustained delivery directly to tumor tissue. Upon FLASH irradiation, the system triggered exosome rupture, resulting in the release of MSA-2 and subsequent activation of the STING pathway, which promoted type I interferon release, dendritic cell (DC) maturation, and reversal of the immunosuppressive TME. *In vitro* studies demonstrated significant DNA damage, immunogenic cell death (ICD), and proliferation inhibition in 4T1 cells following treatment with MEM and FLASH (EM+F). *In vivo* studies on female BALB/c mice demonstrated potent antitumor effects on both primary and distant tumors in a bilateral 4T1 mouse model, improved survival, and induced immunological memory, as shown by resistance to tumor rechallenge. The platform also showed excellent biosafety, minimal systemic toxicity, and synergistic radiosensitization, establishing its promise as a minimally invasive, immune-activating theranostic approach for radioresistant cancers (Figure 5).^73^

**Figure 5:**
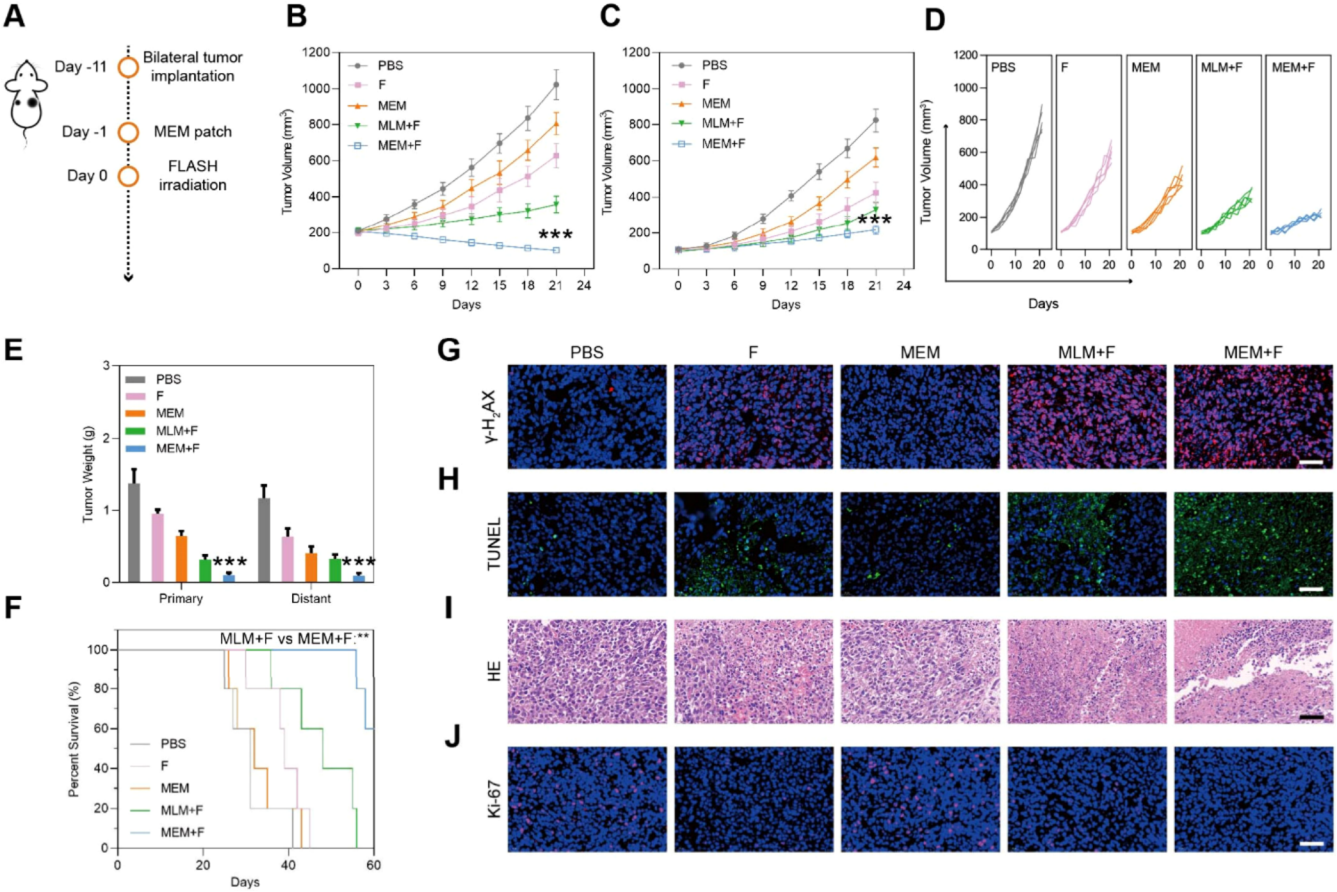
*In vivo* tumor suppression in the bilateral tumor model. (A) Schematic illustration of the experimental procedure. (B) Primary tumor volume. (C) Distant tumor volume and (D) volume in each treatment group (*n* = 5). (E) Tumor weight of primary and distant tumors in each treatment group. (F) Mice survival. Representative images of (G) γ-H2AX staining in the primary tumor. (H) TUNEL, (I) H&E, and (J) *K*_i_-67 staining in the distant tumor. Scale bar: 50 μm. FLASH RT dosage: 10 Gy. Data were presented as the means ± SD. Statistical analysis was performed using one-way analysis of variance (ANOVA; ***P < 0.001). Adapted with permission from ref 73. Copyright 2024, American Chemical Society

A dual-mode ferroptosis-inducing MN platform for effective treatment of TNBC, utilizing a “brake-release and accelerator-pressing” strategy, was introduced by Wang et al. A dissolvable hyaluronic acid–based MN patch was developed to codeliver copper-doped Prussian blue nanoparticles (Cu-PB) and sorafenib (SRF). Upon intradermal insertion, the MNs rapidly dissolve, releasing Cu-PB and SRF into tumor tissues. Under mild NIR irradiation, Cu^2+^ and Fe^3+^ ions are released from Cu-PB, promoting Fenton/Fenton-like reactions that generate reactive oxygen species (ROS), while Cu^2+^ and SRF together inhibit GPX4, disrupting antioxidant defense, which collectively trigger ferroptosis. The MNs demonstrated favorable mechanical strength, skin penetration, rapid dissolution, and laser-triggered drug release. *In vitro*, the Cu-PB+SRF system showed efficient ROS generation, lipid peroxidation (LPO), glutathione (GSH) depletion, and mitochondrial damage, leading to significant cytotoxicity in 4T1 cells under NIR irradiation. *In vivo*, the Cu-PB+SRF@MN (+) treatment in TNBC-bearing mice significantly inhibited tumor growth, prolonged survival, and exhibited high biosafety with no major organ toxicity. Histological analysis confirmed reduced *K*_i_-67 expression, elevated TUNEL staining, and marked GPX4 suppression, consistent with ferroptosis induction. Transcriptomic sequencing further validated alterations in ferroptosis-related pathways, including ROS metabolism, glutathione depletion, and PI3K-Akt signaling.^74^

A multifunctional MN composed of biodegradable HA-MN coloaded with zinc manganese sulfide (ZMS) NPs and the antibiotic sparfloxacin (SP) for the postoperative treatment of TNBC was fabricated by Chu et al. Mechanistically, ZMS releases MN^2+^/Zn^2+^ and H_2_S, disrupting redox homeostasis by depleting GSH and generating ROS, thereby inducing immunogenic cell death (ICD) and mitochondrial dysfunction. Concurrently, SP enhances antibacterial efficacy and inhibits cellular antioxidant enzymes, synergizing with ZMS. The MNs exhibit potent antimicrobial and antibiofilm properties, effectively preventing infections such as MRSA at surgical sites. *In vivo* studies showed that ZS MNs significantly suppressed tumor growth, residual tumor recurrence, and lung metastasis in 4T1 breast tumor-bearing mice, while also accelerating wound healing. They promoted robust immune activation, enhancing the infiltration of CD4^+^/CD8^+^ T cells, dendritic cells, and memory T cells at both local (tumor/lung) and systemic (spleen) levels. The therapy also activated the cGAS-STING pathway, increasing the expression of cytokines involved in antitumor immunity, offering promising approach for postoperative TNBC management.^75^

Weng et al. reported the development of an MN-based dual delivery platform combining gold nanorods (AuNR-PEG) for photothermal therapy and anti-PD-1 peptides for immune checkpoint blockade, aimed at treating early stage BC. The DMN patches were engineered with suitable mechanical strength, efficient skin penetration, and rapid NIR-responsive dissolution. The AuNR-PEG nanomaterial demonstrated strong photothermal heating under 808 nm laser irradiation, effectively ablating tumor cells *in vitro* and *in vivo*. The anti-PD-1 peptide, concentrated mainly at the microneedle tips, activated T lymphocytes and enhanced immune responses. The combined treatment synergistically induced dendritic cell maturation, elevated proinflammatory cytokines (TNF-α, IL-6, IFN-γ), and increased CD8^+^ cytotoxic T cell infiltration in the spleen. *In vivo* studies in 4T1 tumor-bearing Balb/c mice model showed significant tumor suppression, enhanced immune activation, and increased survival in mice receiving MN-based codelivery compared to intramural injection.^76^

Theranostic MNs offer a transformative approach by integrating diagnostic and therapeutic functionalities into a single, minimally invasive platform. These MNs enable early detection, targeted drug delivery, immune modulation, and real-time monitoring, significantly enhancing treatment precision and efficacy. Recent preclinical studies demonstrated their ability to activate immune pathways, induce immunogenic cell death, and suppress primary and metastatic tumor growth with minimal systemic toxicity.

## 7 Microneedle Marvel Or Risk? Unpacking Toxicity and Safety in NPs-Based Breast Cancer Treatments

Transdermal delivery involving NPs is owing to its ability to improve drug solubilization, enhance penetration, and control release; however, safety, biocompatibility, and toxicity must be considered before being utilized in biomedical applications. However, regarding the interactions of NPs with biological systems, there arise concerns regarding toxicity, as NP characteristics that might affect their toxicity include size, shape, aspect ratio, crystallinity, and surface coating.^77^ These properties are highly important in transdermal delivery when a particle needs to cross the skin barrier while minimizing adverse effects such as inflammation or systemic toxicity. NPs with reduced size exhibit enhanced reactivity and cytotoxicity, attributed to their increased surface area-to-volume ratio.^78,79^ The shape and aspect ratio of the NPs have a direct effect on cellular uptake and toxicity. For example, needle-like and high aspect ratio particles exhibit higher toxicity than their spherical counterparts. The aspect ratio affected cellular uptake and cytotoxicity using fabricated mesoporous organosilica nanorods with tunable aspect ratios (2 to 4). Of note would be MORs-3, which was highly internalized into MDR-MCF-7 cells, rendering cell viability below 70% likely because of its fitting size and higher drug delivery efficiency.^80^ Crystalline structures and surface functionalization further modulate toxicity by altering NP stability and interaction with biological systems.^81,82^ Surface functionalization is therefore essential to hinder the production of reactive oxygen species (ROS) and improve the skin’s tolerance toward NPs delivery by inhibiting irritation or allergic responses. Functionalized surfaces enable targeted drug delivery with greater precision in its therapeutic application, minimizing off-target effects. Additionally, customized surface properties enhance the stability of the NPs in biological fluids, preventing agglomeration and allowing for effective delivery. These adaptations increase the safety and efficacy of the NPs.^81,83^ Hence, accurate characterization is critical for the proof of concept of NPs in biomedical applications.

The biocompatibility, biodegradability, and stability of MNs determine their safety and effectiveness in clinical applications. Biocompatibility testing is conducted based on exposure duration, cytotoxicity sensitization, irritation, and intracutaneous reactions for short-term exposure (<24 h). For prolonged use (>30 h), genotoxicity and systemic toxicity tests are essential to ensure the safety of the system over extended periods.^84^ Systemic toxicity and genotoxicity testing for MNs with controlled release systems would be required so that the prolonged exposure to drug/NP-loaded MNs is not harmful; this is also because sustained-release formulations may stay intact with the skin for extended periods.^84,85^ Biodegradability is crucial for MNs, with biodegradable polymers widely used to enable safe degradation and removal from the body while facilitating controlled release through resolution in skin fluid.^86^ Panda et al. prepared FITC-dextran-loaded MNs with PLGA for controlled drug release. MNs prepared from PLGA at different ratios of glycolic and lactic acid had varied degradation rates; PLGA 50:50 degraded faster than PLGA 65:35. The PLGA MNs showed a sustained release profile with a controlled release mechanism influencing both mechanical strength and the drug permeation profile, which demonstrated significant drug permeation through the rat’s skin.^87^ Stability is another crucial aspect, particularly for sensitive therapeutic proteins and peptides. MN stability is evaluated under various storage conditions (−25 to 60 °C), with rigid matrices enhancing stability by reducing molecular mobility and oxygen exposure.

In the study by Choi and Kim, they investigated the biodegradability of polyethylenimine (PEI)-coated MSNs under neutral (pH 7.4) and acidic (pH 5.0) conditions. Compared to uncoated or F-127-coated MSNs, PEI-MSNs exhibited significantly enhanced degradation due to PEI’s pH-buffering effect, which has a favorable pH for silica hydrolysis. The surface coating by PEI facilitated rapid particle breakdown, achieving high degradability (∼83%) even in acidic environments. This was confirmed by inductively coupled plasma optical emission spectrometry, transmission electron microscopy and fluorescence spectroscopy. This study highlights PEI’s role in enhancing MSN biodegradability via its proton-sponge effect, providing insights for designing silica-based carriers with improved degradation in physiological and cellular environments.^88^ Woźniak-Budych and colleagues developed sulfobetaine-stabilized copper oxide NPs (Cu_2_O NPs) functionalized with HA and glutathione (GSH) for cancer therapy. Stability studies in simulated biological fluids (gastric, intestinal, salivary and blood plasma) revealed that Cu_2_O NPs’ behavior, including aggregation and copper ion release, depends on fluid pH, ionic strength and chemical composition. HA-modified Cu_2_O NPs demonstrated higher stability and lower aggregation compared to GSH-modified or unmodified NPs, particularly in blood plasma. Cytotoxicity assays showed greater cancer cell susceptibility to HA-modified Cu_2_O NPs, highlighting their therapeutic potential for target cancer treatment.^89^ Despite the use of biocompatible materials in the fabrication of NPs, they still pose a risk to organs, with toxicity influenced by the properties of the NPs.

The study by De Matteis et al. evaluated the toxicity of anatase and rutile titanium dioxide (TiO_2_) NPs under varying pH conditions and sunlight exposure. Anatase NPs exhibited higher toxicity, driven by greater ionization and ROS generation, especially at acidic pH and in the presence of sunlight. Cellular uptake and mitochondrial dysfunction were more pronounced in MCF-7 cells for anatase NPs. The findings tell about the influence of pH and light on TiO_2_ NPs and underscore the need for careful consideration of factors to minimize dermal toxicity.^90^ Reducing toxicity includes fabricating NPs with biocompatible materials and using light-triggered degradation. Yang et al. developed red light-responsive, diselenide-bridged MSNs coloaded with DOX and methylene blue for synergistic chemo-photodynamic-immunotherapy against BC. Low-dose red light irradiation induced ROS-mediated NP degradation and dual drug release, enhancing effects.^91^

Avoiding the mononuclear phagocytic system remains challenging, as the NPs tend to absorb proteins, forming a protein corona that leads to uptake by the mononuclear phagocytic system.^92^ Nonetheless, emerging strategies like microfluidic synthesis and standardization protocols are crucial for clinical translation. However, continued research into combination therapies, personalized medicine, and improved accessibility can further advance NP-based treatments.^93–95^

MN-based delivery systems are minimally invasive, painless administration, and have potential for patient self-application, representing it as a promising strategy for BC management in low-resource or at-home settings. These systems circumvent the requirement for trained medical personnel, aseptic conditions, and hospital infrastructure, making them particularly advantageous for elderly, disabled, or immunocompromised individuals in remote or underserved regions.^96,97^ In contrast to conventional chemotherapy, which often necessitates intravenous administration and cold-chain storage, microneedle patches can be stored at room temperature and administered with minimal logistical complexity, thereby reducing treatment costs and enhancing accessibility. Moreover, MNDDS have demonstrated potential for localized drug delivery to breast tumors via transdermal routes such as the mammary papilla, achieving higher drug accumulation at the tumor site while minimizing systemic exposure.^32,97^ In conclusion, though NPs and MNs offer promising advances in the transdermal drug delivery system, the safety, biocompatibility, and toxicity of NPs must be carefully evaluated. Advancements in surface functionalization, biodegradability, and stability are highly needed to ensure these systems are effective and safe for long-term usage in clinical settings.

## 8 Evaluation of Repeated Microneedle Use: Safety and Skin Tolerability

Evaluating the safety of repeated MN application is critical for chronic therapies and monitoring. Available evidence suggests that, when used correctly, repeated MN application does not significantly impair skin barrier function or elicit systemic inflammatory or immunogenic responses. In an *in vivo* study, Vicente-Perez et al. investigated the repeated application of dissolving and hydrogel-forming polymeric MN arrays in hairless Crl: SKH1-Hr^hr^ mice. Despite weekly or biweekly applications over several weeks, transepidermal water loss (TEWL) consistently returned to baseline prior to subsequent insertions. Mild erythema observed postremoval resolved spontaneously, and no significant changes were noted in serum biomarkers such as C-reactive protein (CRP), immunoglobulin G (IgG), interleukin-1β (IL-1β), or tumor necrosis factor-α (TNF-α), confirming the absence of systemic inflammation or immune activation.^98^

Similarly, Al-Kasasbeh et al. conducted a clinical study in healthy human volunteers using hydrogel-forming MN patches. TEWL measurements showed transient elevations immediately postremoval, which normalized within hours. Clinical scoring revealed no persistent erythema or skin damage, and systemic biomarkers—including CRP, TNF-α, IL-1β, IgE, and IgG—remained within physiological ranges throughout the study period. These findings collectively affirm the dermal and systemic safety of repeated MN application under controlled conditions.^99^ These findings support the feasibility of MN-based drug delivery for chronic or repeated use in clinical settings

While these findings support the dermal and systemic safety of repeated MN use under controlled conditions, further investigation is needed to assess long-term applications, especially in patients with compromised skin conditions such as eczema or psoriasis. Additionally, safety outcomes may vary depending on MN geometry, application technique, and material composition, which underscores the need for device-specific evaluation in broader clinical contexts.

## 9 Conclusion

The incorporation of NPs into MNs represents a groundbreaking strategy for addressing the challenges found in traditional BC treatments. By enhancing drug delivery through improved bioavailability, targeting capabilities, and reduced systemic toxicity, NPs make significant strides in fighting cancer. Meanwhile, MNs provide a minimally invasive method for localized medication delivery. This integrated system has great potential to enhance the effectiveness of cancer therapies while also paving the way for theranostic platforms that combine both treatment and diagnostic functionalities. NP-based therapies have emerged as the frontier in modern therapeutic approaches, mainly in cancer, enabling drug delivery with unprecedented accuracy and precision. Yet, their clinical translation is hindered by challenges such as complex fabrication processes, scalability issues and unpredictable interactions with biological systems. These limitations, however, provide compelling opportunities to innovate and refine NP design, surface functionalization, and delivery performance.

Ongoing research in MN fabrication techniques particularly advances in micromachining and 3D printing is expected to improve mechanical integrity, optimize matrix-NP compatibility, and streamline mass production. Their ability to effectively encapsulate varied therapeutic agents—including chemotherapeutics—underscores their transformative potential in modern medicine.

To bridge the gap between preclinical studies and clinical applications, future efforts must focus on enhancing NP stability, biodistribution, and therapeutic outcomes while ensuring safety and regulatory compliance. Advancements in preclinical and translational studies, supported by improved *in vitro* and *in vivo* models, would enhance the possibility of bridging the gap between studies in animal and human clinical trials, which will be invaluable. Such developments will be complemented by efforts to improve NP’s stability, dispersibility, and therapeutic effects while maintaining the safety standards. Current limitations include preclinical models that might not predict human responses. Advancements in imaging and tracking technologies, such as fluorescence or magnetic resonance imaging-compatible NPs, would be more useful in understanding MN penetration, NP release, and biodistribution dynamics.

## Supplementary Material



## Data Availability

No primary research results, software or code have been included, and no new data were generated or analyzed as part of this review.

## Notes

Detailed discussion on nanoparticles in breast cancer therapy, targeting strategies, and various MN designs and fabrication methods. Supplementary Figures (S1–S9) illustrate nanoparticle types, targeting mechanisms, MN structures, and *in vivo* outcomes. Table S1 provides comprehensive comparisons of nanoparticle release mechanisms from MNs, and Table S2 provides an overview of preclinical studies of NP-loaded MNs in breast cancer therapy. Additional references with respect to the content are provided in the Supporting Information (PDF)
