# Supplementary material for: Microneedles as Gateways: Smart Nanoparticle Delivery for Enhanced Breast Cancer Treatment
Source: ACS Omega. 2025 Sep 12;10(37):42135–50. doi: 10.1021/acsomega.5c04565 (PMC12461323; doi:10.1021/acsomega.5c04565)
Supplement: Supplementary file 1 [file ao5c04565_si_001.pdf]

Microneedles as Gateways: Smart Nanoparticle Delivery for Enhanced Breast Cancer Treatment

Viola Colaco[1], Deepanjan Datta[1], Ritu Kudarha[1], Abhishek Kumar Singh[2], Namdev Dhas[1*]

[1]Department of Pharmaceutics, Manipal College of Pharmaceutical Sciences, Manipal Academy of Higher Education, Manipal 576104, Karnataka State, India

[2]Manipal Centre for Biotherapeutics Research, Manipal Academy of Higher Education, Karnataka, Manipal, 576104, India

***Corresponding Author**

Dr. Namdev Dhas

Associate Professor

Department of Pharmaceutics

Manipal College of Pharmaceutical Sciences

Manipal Academy of Higher Education (MAHE),

Manipal, Udupi, Karnataka State, India-576104,

Email: namdev.dhas@manipal.edu

Ph: +91-7284087284

SUPPLEMENTARY INFORMATION

S1. Nanoparticles in breast cancer therapy

S1.1. Types of nanoparticles used in breast cancer treatment

Recently, many NPs have been used to deliver the drug to the target site due to their smaller size, shape, surface charge, structure and surface modifications. The specific aim of NPs as an effective delivery system is to provide clinical advantages by augmenting bioavailability, stability and target specificity while reducing side effects, thus supplanting the restriction of conventional therapeutics [1–3]. Besides, NPs enhance drug solubility, ensuring effective delivery of the drug to specific target sites while prolonging circulation time and optimizing pharmacokinetics without compromising the drug stability *in vivo* [4]. The NPs include liposomes, polymeric nanoparticles, solid lipid nanocarriers, nanostructured lipid carriers, mesoporous silica nanoparticles, and gold nanoparticles **(Figure S1)**.

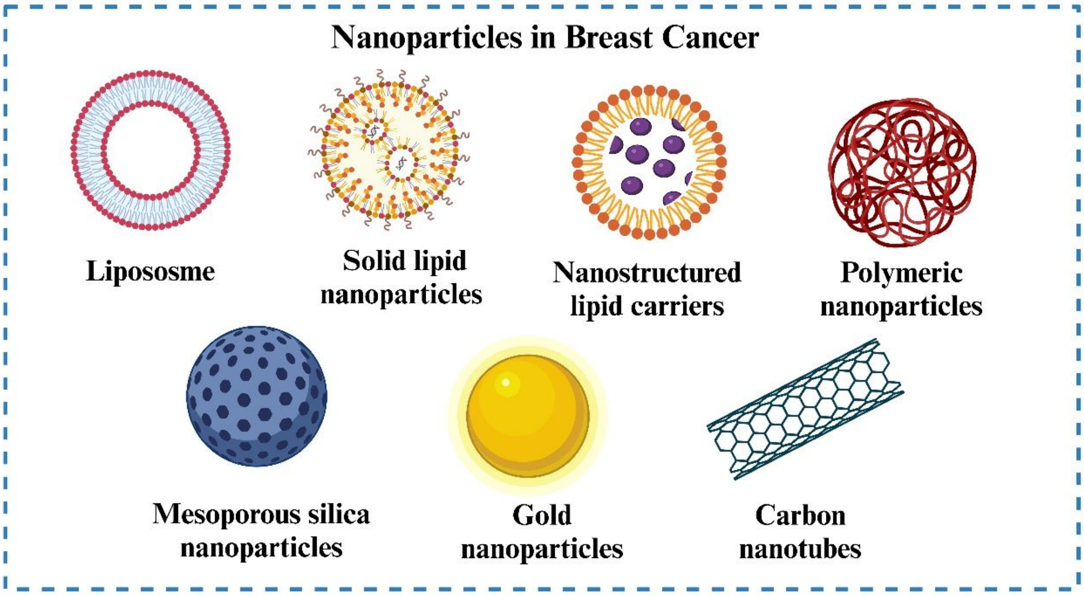

Figure S1. Schematic representation of the types of NPs commonly used for BC therapy. Created using BioRender

S1.1.1. Lipid nanoparticles

Among the NPs, lipid nanoparticles (LNPs) have recently become one of the most promising technologies in BC, taking their full potential into account for improved drug delivery and efficacy. The LNPs comprise several types of lipid molecules, such as ionizable lipids, helper lipids, and cholesterol, that allow drugs to cross cell membranes [5]. These NPs utilize the

enhanced permeability and retention (EPR) effect for preferential accumulation within the tumor tissue, therefore reducing off-targeting and ensuing damage to the noncancerous cells [6]. LNP's have proven to be highly promising in delivering many drug ingredients to prepare drug combinations that would more favourably accumulate in tumors and reduce their systemic toxicity. Ongoing clinical studies are determining its safety and efficacy. Thus far, LNP has emerged as an invaluable tool in the treatment of cancers. Their ability to deliver a spectrum of drugs with decreased side effects makes them candidates to revolutionize the therapy of BC [5,7,8]

S1.1.1.1. Liposomes

Liposomes are spheroidal or multilamellar vesicles formed through self-assembly from diacyl-chain phospholipids in an aqueous solution, creating an amphiphilic bilayer structure comprising hydrophilic heads and hydrophobic tails. They can be prepared from natural or synthetic phospholipids with characteristic particle size, flexibility, fluidity, stability, and charge characteristics of the lipid composition [9]. The hydrophilic moiety bears either a negative charge, a positive charge or a zwitterionic charge. It enhances NP stability due to electrostatic interaction, while the hydrophobic moieties vary in chain length, acyl symmetry and saturation [10].

S1.1.1.2. Solid Lipid Nanoparticles

Solid lipid nanoparticles (SLNs) are spherical colloidal carriers of a solid lipid core matrix, a drug-loaded carrier surrounded by a stabilizing surfactant layer in the aqueous phase. The SLNs include solid lipids, liquid lipids, and emulsifiers; such combinations offer structural stability and functional versatility for SLNs. SLNs demonstrate the ability to encapsulate both hydrophilic and hydrophobic drugs with high entrapment efficiency [11–13]. The release of drugs out of SLNs can be altered by modulating lipid content. With surface modification, SLNs may enable selective drug targeting towards specific locations with tissue barriers, provide stability enhancement, and permit a broader range of applications of SLNs as a drug carrier [11]. The SLNs are actively being studied as an adjunct method to allow bypassing issues arising from more traditional therapies to improve drug delivery and therapeutic efficacy [6].

S1.1.1.3. Nanostructured lipid carriers

Nanostructured lipid carriers (NLCs) were introduced to overcome the drawbacks of SLN [14]. These solid lipid systems consist of solid and liquid lipids, forming an unstructured lipid matrix.

The liquid lipids inhibit crystallization of the lattice, which creates defects in the core of the lipid, thus maximizing loading capacity and solubilization of hydrophobic drugs [15,16]. Studies show that NLCs may be considered a potential carrier for chemotherapeutic drugs, improving stability, pharmacokinetics, and the therapeutic effects of drugs. In addition, some reports indicate that the use of drug-loaded NLCs, observed either alone or in conjugation with targeting moieties, enhanced the values of those inhibiting IC_{50} *in vitro* and *in vivo,* as well as tumor inhibition [17].

S1.1.2. Polymeric nanoparticles

Polymeric nanoparticles (PNPs) are generally made from different polymers and have the ability to control size, shape, and surface charge [18]. PNPs conjugated or encapsulated with drugs include micelles, nanospheres, polymer-drug conjugates, nanogels and dendrimers [4]. Commonly used polymers for PNPs include polylactic-co-glycolic acid (PLGA), polycaprolactone (PCL), polylactic acid (PLA), chitosan, and gelatin [19]. PLGA is an FDA-approved polymer known for biocompatibility and surface modification versatility; PLA is biodegradable and shows slow release from formulations. PCL is a biodegradable polymer suited for long-term drug release and tissue engineering [20].

S1.1.3. Gold nanoparticles

Gold nanoparticles (AuNPs) show characteristic physical and chemical features collectively known as surface plasmon resonance (SPR). Upon illumination by optical radiation, the coherent oscillation of conduction band electrons induces an electromagnetic field in the metallic NPs alongside a dipolar oscillation aligned with the electric field of the light. This effect boosts the radiative properties, such as absorption and scattering, referred to collectively as SPR [21]. Biocompatibility, nanoscale size enabling tumor penetration after systemic delivery, easy functionalization through gold-thiol conjugation chemistry, and adjustability in near-infrared (NIR) light absorption contribute an array of attributes that position AuNPs at the forefront as drug carriers in photothermal therapy (PTT) [22]. These features allow AuNPs to deliver drugs to specific sites, imaging, and PTT, thus making them highly suited for theranostic applications [23].

S1.1.4. Mesoporous silica nanoparticles

Mesoporous silica nanoparticles (MSNs) have gained significant attention due to their uniform, tunable pore size (2-50 nm, as defined by IUPAC), high surface area (>700 m2/g), and large

pore volume (>0.6 cm3) [24]. These inorganic nanoplatforms, typically 50-300 nm in diameter, allow for independent surface functionalization, both internally and externally, and feature adjustable pore structures based on surface selection. Their unique release mechanism and ordered pore arrangement make MSNs highly promising carriers [25].

S1.1.5 Carbon nanoparticles

Carbon nanoparticles (CNPs) include carbon nanotubes (CNTs), graphene, and quantum dots (QDs), each exhibiting unique properties like high stability, conductivity, mechanical strength, and biocompatibility. Because of sp^2 hybridization, they are very hydrophobic [26]. CNTs are effective drug carriers, which allow non-invasive penetration across biological membranes. Drug molecules can be loaded onto CNTs via covalent and non-covalent interactions; however, they balance stability and control release challenges. Encapsulating drugs within CNTs and utilising stimuli like heat or light-enhanced target delivery. Graphene and graphene oxide (GO), with their large surface area and pi-electron interaction, are excellent platforms for drug delivery. CQDs exhibit size-dependent luminescent, optical and electronic properties, promising in biosensing and biomedical applications due to their solubility, low toxicity and stability [27,28].

S2. Targeting strategies to improve the efficiency of NPs in BC

S2.1. Passive targeting

Passive targeting leverages the enhanced permeability and retention (EPR) effect, where NPs accumulate in tumor tissues due to permeable vasculature and impaired lymphatic drainage in the tumor microenvironment (TME), which enhances drug delivery while avoiding rapid renal clearance, increasing therapeutic efficacy and reducing systemic toxicity [29,30]. The EPR effect enables the accumulation of therapeutic agents in tumors by exploiting the defective vasculature and poor lymphatic drainage of tumor sites. This passive targeting increases drug concentration in the tumor, improving efficacy and minimizing system toxicity [30]. Tumor-induced neovascularization leads to vessels with large pores and enhanced NP permeability [29]. However, the heterogeneity of human tumors, including variations in pore distribution, hypoxia and extracellular matrix (ECM), can reduce the effectiveness of EPR in targeting cancer cells [31]. The NPs sized 10-100 nm enhance the EPR effect, avoiding rapid renal clearance, which boosts tumor drug delivery and reduces systemic toxicity [32]. Circulation time is influenced by surface chemistry and charge, with hydrophilic NPs being preferred. Water-soluble polymers like PEG are grafted onto nanocarriers to achieve this, preventing aggregation and non-specific

interactions. Clinically approved passively targeted nanocarriers include Doxil, Onivyde, Abraxane, Genexol-PM, Myocet, and DaunoXome [33].

S2.2. Active targeting

The rapidly growing tumors result in abnormal expression of specific antigens or receptors on the endothelial cells of tumor vasculature as compared to their expression in normal tissues. Surface modification of NPs with antibodies or ligands that target these markers improves the accumulation efficiency in the tumor while enabling the achievement of target delivery of drugs [34]. Active targeting involves ligand-receptor recognition using different ligands, including peptides, proteins, nucleic acids, antibiotics, carbohydrates, and small molecules such as FA and transferrin. Ligand selection depends on the target overexpression in a specific cancer type [29]. Active targeting can also occur through drug release triggered by pathological tumor conditions like low pH or aberrant enzyme expression. Targeting sites include tumor vasculature, stroma, cells and the immune system, though these categories can overlap. For instance, $\alpha_v\beta_3$ integrins are found in both the tumor vasculature and specific BC cells [31].

The challenges of NP-based therapies include the complexity of development, knowledge about biological interactions, limited clinical translation and lack of standard regulatory frameworks [35]. Despite their benefits, NP-based therapies face challenges, including safety concerns, complex distribution, manufacturing hurdles, and high production costs. NPs face particular challenges that are divided into study-design-related, technological, and biological. The study design challenges of the NP therapy include the study sizes, time, and model selection involved in clinical trials. Human responses have rarely been matched by animal or cell models because it is extremely challenging for humans to imitate natural responses. Technological challenges encountered in NP-based therapies relate to scale-up synthesis, equal optimization, and predicting performance, which is critical to the ultimate success of treatment in a clinic [36]. The biological challenges are mainly limited routes of administration, changed biodistribution, difficulties in crossing the barrier, degradation, and toxicity. Biological barriers pose challenges in crossing NP transport, including tight junctions, physiological defences, and secondary barriers. The parameters governing NP transport are size, charge, shape, and surface modification, all of which may change due to the formation of the protein corona [37].

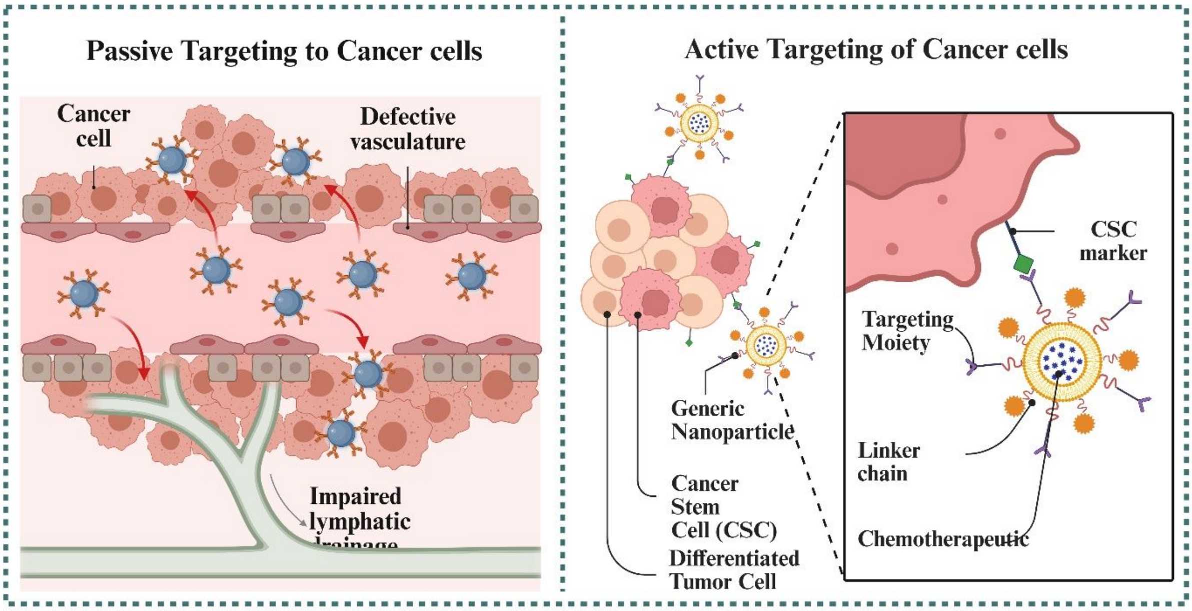

Figure S2. Schematic illustration of strategies that enhance cancer treatment using NPs: passive targeting and active targeting drug release. Created using BioRender.

S3. Types of MNs unveiled as a carrier for NPs

MN patches, though, have a versatile design based on the delivery method and drug characteristics, generally consist of micron-scale tapered needles arranged in arrays on a base substrate and backed by an adhesive patch for skin applications [38,39]. MNs can be classified based on delivery approaches, as follows: The different types of MNs, along with their release mechanism, advantages and limitations, have been listed concerning BC studies in **Table S1** and **Figure S3.**

S3.1. Solid MNs: "Precision without payload: Solid MNs as a smart skin interactors"

Solid MNs are drug-free, microscale, tapered projections composed of single materials, used to puncture the SC and create transient microchannels in the skin [40]. These pores enhance transdermal drug delivery by allowing subsequent applications of conventional formulations, improving bioavailability [39,41]. This type of MN uses a "poke-and-patch" strategy, which was first demonstrated using silicon MNs offering precise microfabrication capabilities but presented limitations including high cost, fragility, complex processing, and biocompatibility concerns. Consequently, alternative materials – biocompatible, robust, and easily fabricated – have been explored to enhance the delivery of proteins, hormones and vaccines [38]

S3.2. Coated MNs: "a thin layer of innovation: Coated MNs for optimized delivery"

Coated MNs are solid MNs with a drug layer applied to their surface, enabling rapid drug release upon skin insertion through the fast dissolution of the coating. Efficient drug delivery depends on uniform coating, influenced by formulation properties such as wettability, solubility and mechanical strength. The coating techniques include dip-coating and layer-by-layer assembly, allowing precise control of drug loading and release kinetics. To enhance precision, apparatuses for controlled dipping have been developed[38,42]. This type of MNs uses "coat and poke release" upon insertion, the coating dissolves, releasing the payload. Coated MNs have been employed for transdermal delivery of vaccines[43], proteins[44], hormones and peptides[45]

S3.3. Hollow MNs: "Micro pathways to efficiency: Hollow MNs for controlled transport"

Hollow MNs feature an empty core with a tip opening, enabling pressure-controlled, rapid delivery of high molecular weight bioactives and large drug doses. These MNs can hold larger drug volumes and allow precise control over the delivery rate by adjusting the applied pressure. Hollow MNs deliver drugs via a poke-and-flow approach[38,39]. Beyond liquid formulations, hollow MNs effectively deliver nanoparticles such as PLGA[46], liposomes, and mesoporous silica nanoparticles [47]. Studies show that hollow MNs enhance antigen delivery and immune responses compared to other MN types, highlighting their potential in vaccine and nanoparticle-based therapies [48].

S3.4. Dissolvable MNs: "Vanishing barriers: Dissolving MNs for seamless drug delivery"

Dissolvable MNs are fabricated from biocompatible and biodegradable polymers such as hyaluronic acid (HA), polyvinylpyrrolidone (PVP), polyvinyl alcohol (PVA), carboxymethylcellulose (CMC), chitosan, poly(lactide-co-glycolic acid) (PLGA) and polylactic acid (PLA) that encapsulate drugs and dissolve in the skin after insertion. They follow a "poke-and-dissolve" mechanism and are ideal for delivering low drug doses [38,49]. Biodegradable MNs release drugs over extended periods via diffusion and polymer erosion. To improve drug loading and mechanical strength, advanced designs like double-layered, pedestal, and separable arrowhead MNs were invented [50]. While dissolvable MNs offer low-cost, waste-free delivery without pumps or patches, they face limitations such as low strength, limited drug capacity, and formulation challenges[38].

S3.5. Hydrogel MNs: "Soft yet strong: Hydrogel-based MNs for advanced therapeutics"

Hydrogel MNs are composed of swellable polymers that absorb interstitial fluids upon insertion, forming a 3D hydrogel network that enables controlled drug release from an attached reservoir [38,51]. These MNs leave no residue post-removal and allow delivery of both small and large molecules. Materials like PMVE/MA, PEG, and silk fibroin are commonly used. They follow a "poke and release" mechanism for drug delivery. They can also enable on-demand drug release via external stimuli (e.g., light) and are typically fabricated using micromolding techniques [52].

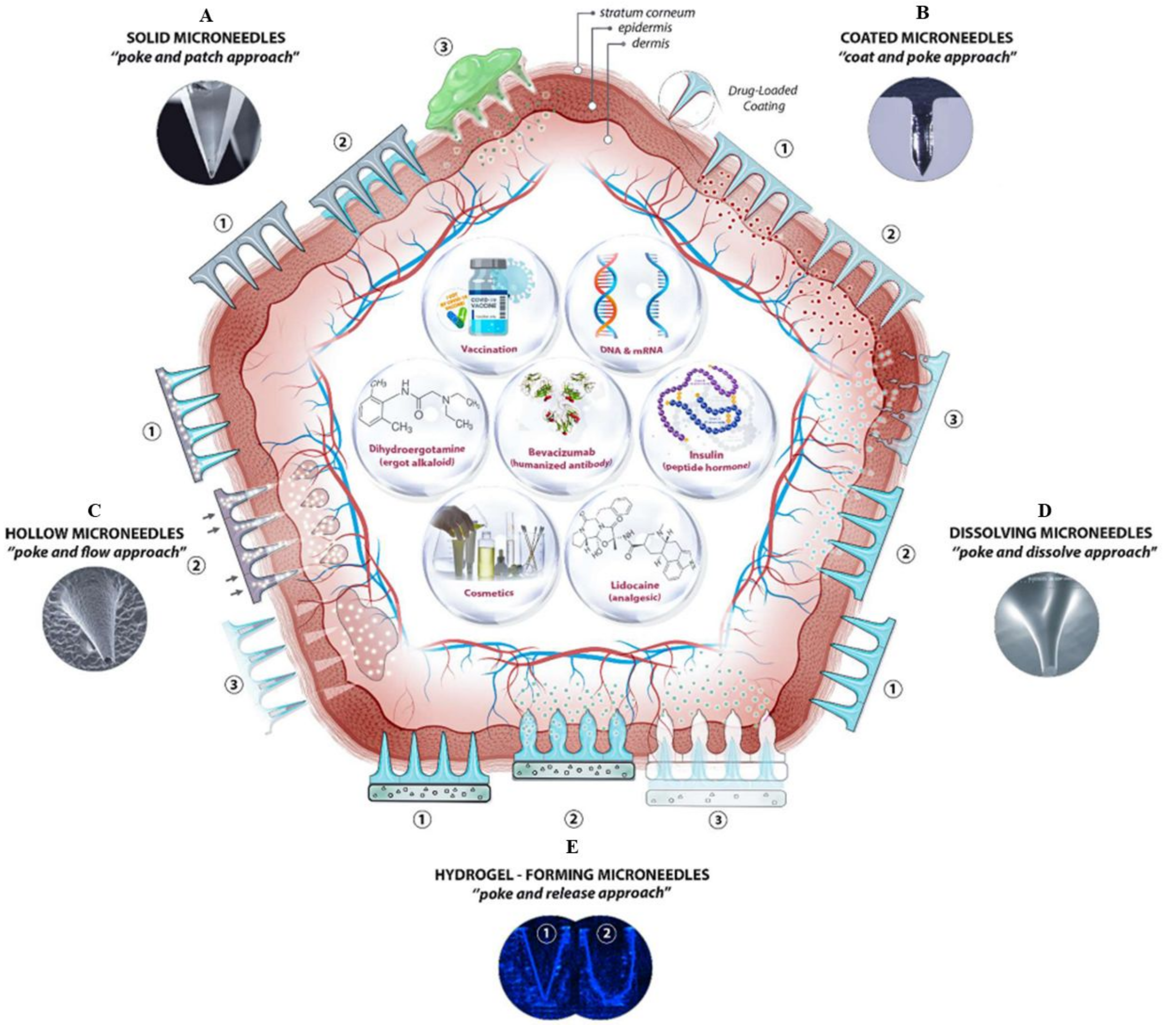

Figure S3. A schematic illustration of MN-based drug delivery methods depicting a cross-sectional view of the upper layer of the skin. The approaches include (A) solid MNs, (B) coated MNs, (C) hollow MNs, (D) dissolving MNs, and (E) hydrogel-forming MNs. Each delivery system is presented as a sequential process, detailed in three distinct steps, numbered 1 to 3. Adopted with permission from [53]. This is an open-access article, available under the terms of the Creative Common CC-BY license. Copyright 2021, Muhammet Avcil.

Table S1. Mechanism of nanoparticles release from MNs

Type of MNs	Mechanism of release	Fabrication techniques	Advantages	Limitations	Ref
**Solid MNs**	Poke and patch<br>Pore-forming pretreatment	Micromolding and wet etching	Ease of fabrication<br>Suitable for delivering proteins, hormones and vaccines	Two-step administration required<br>Inexact dosing<br>Fragility and cost (especially for silicon-based MNs)	[54–56]
**Coated MNs**	Coat and poke release<br>The drug is coated on the surface of the MNs. Upon insertion, the coating dissolves, releasing the payload	Micro molding<br>Centrifugal lithography	It avoids bioactive material degradation during manufacturing<br>Enables uniform and controlled drug delivery<br>Useful for biomarker sampling	Challenges in achieving uniform coating and stability<br>This may result in a low penetration ability<br>Small drug doses may reduce mechanical strength	[52–54]
**Hollow MNs**	Poke and flow release<br>Therapeutic ingredients are delivered through hollow channels by	MEMS, wet chemical etching, deep reactive ion etching of silicon, deep X-ray photolithography	Allows controlled dosing	Complex construction and susceptibility to clogging	[52,57,58]

	diffusion, pressure or pumps		Can be integrated into lab-on-chip devices<br>Suitable for delivering proteins, vaccines, mRNA and diagnostics	Risk of drug leakage and structural fragility<br>Larger tip diameter leads to poor insertion	
**Dissolvable MNs**	Poke and dissolve mechanism<br>Biocompatible polymers dissolve upon exposure to interstitial fluid, releasing the payload	Micromolding	Biodegradable and cost-effective material<br>Eliminates biohazardous waste	Penetration abilities can be inconsistent<br>Low mechanical strength	[54,59,60]
**Hydrogel-forming MNs**	Poke and release mechanism<br>Swellable polymers absorb interstitial fluid, creating conduits for drug delivery or diagnostic sampling	Micromolding<br>Casting, electrospinning	Responsive delivery based on physiological signals<br>Fine-tuned delivery (time from minutes to days)	Limited drug doses and low mechanical strength<br>Requires significant advancements for commercial viability	[54,61]

S4. A brief overview of the MN device

The design of MNs is complex and depends on many parameters, like optimal skin penetration, functionality, and comfort for the patient [62]. The fabrication process of MNs requires a set of techniques and parameters to drive efficacy, safety, and usability. A set of parameters, however, is implemented in the design of MNs, such as length, needle-to-needle spacing, tip diameter and tip spacing, aspect ratio, and needle geometry. Structural design tips, notably diameter, aspect ratio, and height, will influence the performance of the MNs.

S4.1. Structural design of MNs

S4.1.1. Geometry

The morphology of MNs significantly affects the penetration depth due to its association with drug loading capacity, stress distribution, and mechanical performance [63]. The results and computed data also show that with polygon-based MNs, a linear exhibition is demonstrated between the mechanical strength and number of vertices [52,53]. The triangular and square bases have deeper insertion depths than the hexagonal shape, with acute edges presented therein. Hence, more penetration into the skin could be achieved [64,65]. Li et al investigated transcutaneous immunization with dissolving MN arrays of different geometries and delivery of ovalbumin (OVA) delivery. DMNs were fabricated via a two-step molding process using OVA-loaded dextran solution. The geometry assessed included cone, cone-cylinder, hexagonal pyramid, and rectangular pyramid. Cone-shaped DMNs showed the highest drug-loading capacity (approx. 97%) due to their larger volume and effective encapsulation, alongside superior penetration (approx. 95%), and dissolution (approx. 80%). In contrast, cone-cylinder DMNs exhibited poor mechanical strength, resulting in low drug loading and penetration efficiency of approximately. 40%. Rectangular and hexagonal pyramid DMNs showed moderate performance in both drug delivery and mechanical properties [66] **(Figure S4 A).**

S4.1.2. Tip diameter and length

The diameter of the needle tip affects the force required for skin penetration. Smaller tip diameters (around 5 μm) generally require less force compared to larger tips (up to 75 μm). The aspect ratio, defined as the height of the MN relative to its base width, plays a significant role in mechanical stability and penetration capabilities. Optimizing this ratio is essential for effective skin penetration [62,67]. Smaller tip diameters reduce penetration force, and higher aspect ratios facilitate insertion but may compromise structural stability. While taller MNs

improve penetration depth, they can increase pain and bleeding, necessitating a balance in height to achieve effective delivery with minimal discomfort. For transdermal applications, it is essential to consider the structure of human skin, comprising of SC (10-20 µm), epidermis (100-150 µm) and dermis (3-5 mm) [52]. The length of MN typically ranges from 10 to 1000 µm. MN penetration efficacy is influenced by the needle length, as studied by Verbaan et al., where MNs with a base diameter of 300 µm and length of 300 µm failed to pierce the skin, while lengths above 550 µm achieved successful penetration [68] **(Figure S4 B)**.

S4.1.3. Needle-to-needle spacing

The skin's topographical diversity allows significant deformation before penetration. High-density MN arrays (>500 needles/cm^2) require increased energy and force for a puncture, leading to greater patient sensation and a potential need for more substantial devices. Larger, longer, and denser needles create larger and more crowded pores, enhancing drug diffusion [53]. However, considering the relationship between needle length and discomfort, several tactics can be used to increase penetration depth, such as a more potent force of application or a bigger needle-to-needle spacing [52]. Shu et al. evaluated the interspacing in MN using finite element analysis (FEA) to assess its impact on MN insertion and skin response. The results showed that sparse MN interspacing > 0.5 mm pitch improves skin penetration efficiency by allowing better needle-skin contact, with minimal differences in initial force and strain distribution across pitches of 0.5-1.75 mm. Conversely, dense interspacing (< 0.5 mm) increases initial penetration force, reduces efficiency, and shows significant strain and gaps around middle needles due to the "bed of nails effect". Spare interspacing effectively mitigates this phenomenon, enhancing overall MN insertion mechanics **(Figure S4 C)** [69].

S4.1.4. Aspect ratio

Aspect ratio is defined as the ratio of MN height to its base width, significantly influencing insertion efficiency and mechanical strength. MN with a higher aspect ratio facilitates easier skin penetration; however, increasing the aspect ratio by elongating the MN height also elevates the failure force. Conversely, reducing the aspect ratios by widening the base enhances the mechanical strength of the MNs [62]. Choo et al. examined the impact of aspect ratio on 3D printing accuracy and mechanical properties of MNs. Using a fixed height of 1300 µm and varying base sizes (500, 330, 250 and 200 µm), aspect ratios of 2.6:1, 3.9:1, 5.2:1 and 6.5:1 were analyzed. The results showed that the printed base dimensions closely matched the input value across all aspect ratios (t-test, $p > 0.74$), and the tip diameter remained consistent

irrespective of the aspect ratio [70] **(Figure S4 D)**. In another study by Krieger et al., they fabricated MNs with aspect ratios of 3:1, 4:1 and 5:1 using a 25 µm layer height for a smooth surface. Height reductions were more pronounced at smaller input heights and higher aspect ratios, with a 51% reduction observed for 4:1 needles at 600 µm input height. Tip radii range from 20 to 40 µm, decreasing with reduced height, while needle straightness was maintained. Optimized conditions (4:1 aspect ratio, 25-µm layer height and 1.5 mm spacing) facilitated the production of sub-millimetre MNs using a resin-based "Print and Fill" approach [71].

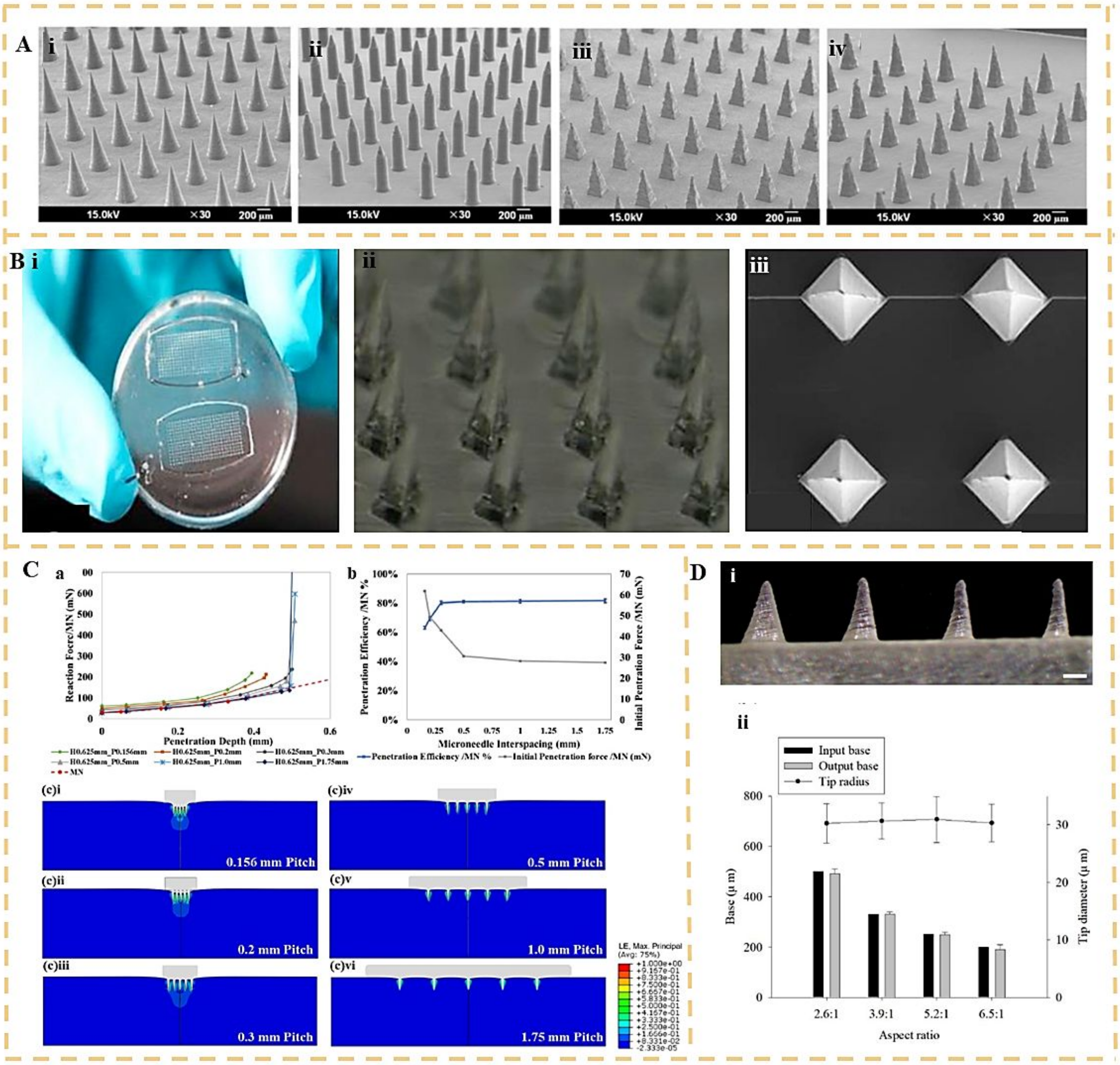

Figure S4. A) SEM images of DMNs: i) Cone, ii) Cone-cylinder, iii) Rectangular pyramid, and iv) Hexagonal pyramid. All DMNs were fabricated using master molds. Adapted with permission from [66]. Copyright 2020, Yingying Li. B) Images of the master mold (i) used for preparing the secondary PDMS mold, (ii) Cross-sectional image of the secondary PDMS mold, and (iii) Scanning electron microscopy of blank DMNs. Adapted with permission from [72].

Copyright 2019, Shubhmita Bhatnagar. C) Evaluating the influence of MN interspace on penetration efficiency: a) the reaction force per MN versus penetration depth for MN arrays with varying interspace (h = MN height, p= pitch or center-to-center distance between MNs, MN (red dashed line) = an individual MN without a base), b) Penetration efficiency and the initial penetration force per MN relative to MN interspacing, ranging from pitch of 0.156 mm (where MN bases are in close proximity) to 1.75 mm, and c) Visualization of strain distribution at the point of maximum penetration for MN arrays, with (c)i 0.156 mm, (c)ii 0.200 mm, (c)iii 0.300 mm, (c)iv 0.500 mm, (c)v 1.00 mm, and (c)vi 1.750 mm pitch respectively. Adapted with permission from [69]. This is an open-access article, available under the terms of the Creative Common CC-BY license. Copyright 2021, Wenting Shu. D) Effect of MN aspect ratios on 3D printing: a) Stereomicroscopic images of MN with an input height of 1300 μm and a 500 μm base, exhibiting aspect ratios of 2.6:1, 3.9:1, 5.2:1 and 6.5:1. b) The dimensions of the input base, output base, and tip diameter according to the aspect ratio (n = 7, scale bare = 1.0 mm). Adapted with permission from [70]. This is an open-access article, available under the terms of the Creative Common CC-BY license. Copyright 2022, Sangmin Choo.

The MN structural design greatly impacts their performance in transdermal drug delivery. key parameters, including geometry, tip diameter, length, needle spacing and aspect ratio, influence penetration efficiency, drug loading, and mechanical stability. The cone-shaped mode of MNs optimizes for drug loading and skin penetration; maximized aspect ratios and less needle spacing assure efficiency and diminish discomfort. A balanced consideration of all these factors enables the development of efficient, safe, and patient-friendly MN design.

S5. The art of MN fabrication

Various techniques are available for microneedle fabrication, including direct manufacturing and mold-based methods, the latter being advantageous for cost-effective large-scale production. The design of the microneedle—specifically the shape, length, and tip width—must be optimized to ensure effective skin penetration and minimize insertion force. Additionally, MNs must exhibit adequate mechanical strength to penetrate the skin without deformation or breakage[52,73]. Achieving this requires careful consideration of the material properties, microneedle geometry, and targeted skin depth. Fabrication techniques are categorised based on the technologies used [74]

S5.1. Fabrication of polymeric MNs using solvents

S5.1.1. Micromolding

Micromolding is a widely used technique for fabricating MNs relying on a negative mould with cavities for needle formation materials such as natural and synthetic polymers ceramics, and hydrogels are filled into the mould followed by a curing process, like solvent evaporation, cross-linking or solidification. The method is cost-effective and reproducible, making it suitable for large-scale production. Micromolding allows the development of various MN structures, including composite multi-regional and core-shell designs; however, challenges such as mould filling due to surface tension limitation, information complex structures and the mold-filling can affect the process [52,74].

S5.1.2. Atomized spraying to fill molds/ Spray deposition

Atomized spraying is an alternative to centrifugation or vacuuming in micromolding, improving scalability for continuous manufacturing. By spraying atomized droplets into micromolds, air pockets are reduced, improving the accuracy and mechanical integrity of MNs [75]. This method employs a nozzle connected to an air source and liquid formulation to fill PDMS molds for dissolving MNs fabrication, followed by drying at room temperature, producing laminate-layered and horizontally layered dissolving MNs, and circumventing restrictions in mass production and geometry. Challenges related to surface tension and viscosity during the filling of the mold are also addressed by this method [52].

S5.1.3. Droplet-born air blowing (DAB)

In the DAB method, a polymer solution is dropped in between two plates, and the upper plate is brought up, stretching the droplet into MNs. The air blowing then dries off the leftover water and thus stabilizes the shape. The dimensional control of MN, its concentration, and the amount of drug loading has been made possible. Kim and co-workers devised the DAB technique for the lithography-based drawing of MNs at low temperatures and under low-pressure conditions such that neither UV radiation nor heat inactivates the drugs [76]. Another success story is that of insulin-based dissolving MNs made using this technique, which has been shown to lower blood glucose levels significantly in diabetic mice [57,74].

S5.1.4. Pulling pipettes

Hollow glass MNs are prepared by softening the glass and then pulling it using a micropipette puller. The programmable pullers allow for reproducibility of this method, but it is only suitable for hollow glass MNs. They have been shown to deliver bolus insulin for type I diabetes and to inject fluids into the skin. In addition, these glass MNs made by this method were

functionalized for intracameral drug delivery in a less invasive manner. In such a manner, 6-aminoquinolone, and Rose Bengal have been successfully delivered to the eye [57,74].

S5.2. Microelectrochemical system (MEMS)

MEMS technology allows for the preparation of micron-scale, sophisticated MN structures, both hollow and solid and dissolvable MNs. MEMS makes use of integrated circuit technology and involves three major processes: deposition, patterning, and etching [74]. The deposition is a process of forming ultra-thin layers on a substrate by either physical vapor deposition (PVD) or chemical vapor deposition (CVD) [74]. Patterning uses the lithography approach, photolithography being very predominant, to reproduce MN designs from photosensitive substrates to successfully obtain precise geometry with a source of UV radiation, as described in references [77].

S5.3. Laser cutting

Laser cutting employs an infrared laser guided by AutoCAD software to shape metallic sheets into MNs, typically using materials like stainless steel or titanium. This process creates in-plane MNs, which are then bent at 90° and electropolished to refine tip sharpness and geometry [74]. Laser-based techniques, including laser ablation and electropolishing on MN molds, allow for the production of solid MN arrays with customizable designs accommodating single or two-dimensional geometries [52,57].

S5.4. Laser ablation

Laser ablation, also known as photoablation, is the process of removal of material from a solid surface with high-power laser beams by pulsed or continuous wave lasers when the intensity threshold of material ablation is attained. It is an accurate, flexible process to manufacture MNs from metal to polymer in nano to micro-scale applications. Different lasers, such as CO_2, UV excimer, and femtosecond laser, can effectively shape MNs as ablation happens within nanoseconds. Thermal effects caused by ablation will be seen in the structure of the MN, causing crack or lowering the fatigue resistance [52,57].

S5.5. Drawing-based methods

Drawing-based techniques use several forces, including mechanical, adhesive, electrostatic and centrifugal, to form the materials into MNs that are then cured. They are low-cost, can be produced in large numbers, and yield mostly simple geometries like conical or hollow needles,

but are limited to complex geometries. Mechanical force drawing is similar to the original glass micropipette techniques and yields hollow glass MNs that have proven to be very effective [52,78]. Contact drawing employs adhesive forces between the material and the drawing device. Subsequent steps of solidification include solvent evaporation or ionic crosslinking air-blowing techniques in this method may allow a soft shaping of polymer droplets without UV or heat, thus providing precise control for the preparation of dissolved MNs with accurate drug delivery [52]. Electro-drawing (ED) is an electrohydrodynamic (EDH) force-driven process to attract polymer droplets in a non-contact manner [79].

Table S2. Overview of MNs containing NPs for drug delivery in BC

Therapy	Drug/ Active ingredient	Nanoparticle	Microneedles	Fabrication techniques	*In vitro* cell line studies	*In vivo* studies	Outcome	Benefits	Limitations	Ref.
Chemotherapy	Fenretinide	Ethosomes	DMNs containing PVA and PVP	Casting method	-	Female SD rats	The MN system achieved prolonged high levels and higher drug concentrations at the application site, reducing systemic side effects	The formulation showed enhanced solubility and skin penetration of Fenretinide, with sustained drug release up to 48 h, and MNs showed optimal mechanical strength for skin insertion	Although plasma concentration is discussed, more detailed PK parameters are not presented	80
Chemotherapy	Atorvastatin	Pumpkisomes	DMNs containing PVA/sodium carboxymethyl cellulose matrix	Micromolding	MDA-MB-231 cells	Female Swiss albino mice	The ATV-PUMP system demonstrated selective cytotoxicity, improved cellular uptake and superior anti-migratory effects in cancer cells. The DMNs exhibited fast	The preparation showed enhanced anticancer activity of atorvastatin, Fast-dissolving MNs with good mechanical strength and skin-piercing ability and effective localized delivery with minimal systemic toxicity	Further PK/PD studies are required for clinical translation	81

							dissolution, sufficient mechanical strength and effective skin penetration, ensuring localized delivery and significant anticancer efficacy in BC model without toxicity to other organs.			
Chemotherapy	Anastrozole	PNPs	MNs containing sodium hyaluronate, PVA cold, PVP K30 and kollidon V4 64	Two-step casting method	-	-	The MNs demonstrated enhanced permeability and adequate mechanical strength, supporting the viability of the transdermal route of drug administration	The nanoparticles achieved high entrapment efficiency and uniform size, demonstrated complete drug release in 3.5 hrs and the PVA-based MNs showed superior mechanical strength and moisture stability	*In vitro* cell line and *in vivo* animal study data is lacking	82
Chemotherapy	Artesunate	SLNs	DMNs containing	Micromolding	MCF-7 cells	-	Incorporating ART-SLNs into	The formulation demonstrated	No *in vivo* pharmacokinet	83

			PVP K-10, HPMC K4, PVP and gelatin	technique with positive pressure-assisted casting			dissolving MN arrays improved ART delivery and cytotoxicity against BC cells. additionally, the transpapillary MNs provide a proof-of-concept for direct delivery to BC cells	effective transpapillary delivery, showed potential for cancer cell targeting	ic or biodistribution data provided	
Chemotherapy	Resveratrol	NLCs	Hollow microneedle array	-	MDA-MB-231 cells	Female Sprague Dawley rats	The Resveratrol-loaded NLCs with MNs significantly enhanced transdermal delivery, breast tissue targeting, cellular uptake and improved therapeutic efficacy	Resveratrol-NLCs showed improved cytotoxicity, enhanced pharmacokinetic profile, localized drug accumulation in breast tissue and potential for prolonged release, releasing dosing frequency and better patient compliance	Study is limited to preclinical evaluation, skin retention data suggesting need for optimization	84
Chemotherapy and	PTX and anti-PD-L1 antibody	Albumin NPs	MNs containing HA	Multistep centrifugation method	4T1 cells	Balb/c mice	The system enables localized, GSH-responsive co-delivery of	Enhanced transdermal delivery efficiency confirmed *in vivo*, superior		85

immunotherapy							chemo- and immunotherapeutics, enhancing antitumor immunity and reducing systemic toxicity	therapeutic effect compared to intratumoral injection and showed reduced effects of chemotherapy and immune-related adverse events		
Chemophotothera py and immunotherapy	DOX and anti-PD-L1 antibody	Black phosphorus quantum dots	DMNs were fabricated with PVP K90 (arrowheads) and PVP K30 and PVA 1788 (supporting base layer)	Two-step micromolding process using layered casting and vacuum filing	4T1 cells	4 T1 primary tumor-bearing and B16F10 melanoma-bearing mice	Microneedle patches enabled deep intratumoral delivery, inducing PTT/PDT-mediated immunogenic cell death, enhancing effector T cell infiltration, and reducing recurrence and metastasis	It showed enhanced effector T cell infiltration and reduced immunosuppressive cells, reduced lung metastasis and eliminated residual tumor post-surgery	Pharmacokinetic data is not discussed	86
Immunotherapy	STING agonist MSA-2 (MEM)	Exosomes fusion liposomes	Porous MNs fabricated from cross-linked methacryloyl porous GelMA embedded with EM (MEM)	Photopolymerization using blue light	MCF-10A	Female BALB/c mice	The EXO-MN combined with FLASH radiotherapy enhance MSA-2 delivery, effectively	Enabled controlled release of MSA-2 under FLASH irradiation via oxidative stress-triggered EXO rupture, effectively	Pharmacokinetic and biodistribution data are not reported	87

							overcoming the radioresistant TME through STING cascade activation-mediated immunotherapy.	activated the STING pathway *in vitro* and *in vivo* and the combination of MEM and FLASH showed strong antitumor effects at both primary and distant tumor sites		
Immunotherapy	Bacillus Calmette–Guérin polysaccharide nucleic acid	Mannosylated chitosan NPs	DMNs fabricated with chitosan and PVP	Micromolding	4T1 cells	BALB/c mice	The MCS NPs loaded DMNs targeted DCs, promote their maturation, and elicit robust antitumor immune responses	Enhanced tumor antigen recognition and immune response with adjuvant use and presents a promising clinical strategy for immunotherapy of aggressive tumors	Mechanistic pathways of DC activation and antigen presentation not fully explored	88
Immunotherapy and antibacterial therapy	Sparfloxacin	Zinc-manganese sulfide NPs	HA-based DMNs	Micromolding	Methicillin-resistant Staphylococcus aureus and 4T1 cells	4T1 tumor-bearing mice	ZMS NPs-MN effectively prevented TNBC recurrence by combining ROS-mediated immunogenic cell death, Mn^{2+}-induced cGAS-STING activation,	Promoted immunogenic cell death (ICD) and increased immune-related cytokines, significantly enhanced immune cell infiltration in tumor, lungs and spleen and no	Lacks pharmacokinetic data and possible immune overstimulation or off-target effects not investigated	89

							and antimicrobial action, promoting antitumor immunity, wound healing, and reduced metastasis	systemic toxicity observed		
Immunotherapy and Photodynamic therapy	Zinc Phthalocyanine and Anti-CTLA4 antibody	Dextran NPs	MNs matrix fabricated using methacrylated HA, N,N′-methylenebisacrylamide and irgacure	Micromolding	4T1 mouse breast tumor cell line	Female BALB/c mice	The MN assisted delivery system co-loaded with a photosensitizer and anti-CTLA4 antibody in acid-responsive pendant acetal modified dextran NPs enabled effective localized photodynamic-immunotherapy in tumor models	Enabled painless epidermal penetration and local drug release through swelling in interstitial fluid, demonstrated significant tumor growth suppression in 4T1 mouse model and PDT induced local immune activation, enhanced by CTLA4 checkpoint blockade	Pharmacokinetic and biodistribution data are not reported and potential variability in drug release based on TME pH not addressed	90
Phototherapy and sonodynamic therapy	$BiVO_4$ Schottky heterojunction, glucose oxidase	$Bi/BiVO_4$ Schottky heterojunction	MNs fabricated using PVP, PVA and sodium hyaluronate	Multilayered vacuum-assisted micromolding technique	4T1 and L929 cells	Female BALB/c mice	The system demonstrated high therapeutic efficacy by enhancing phototherapy and	Enabled controlled "on–off" release of glucose oxidase and dially trisufide under US/NIR irradiation, demonstrated	Detailed pharmacokinetic and biodistribution data is lacking, and potential	91

	and dially trisufide						sonodynamic therapy, controlled drug release under US/NIR irradiation, CT imaging capability, and synergistic tumor therapy via starvation and gas mechanism.	effective *in vitro* and *in vivo* multimodal therapy combining phototherapy, SDT, starvation, and gas therapy and it is a promising platform for BC CT imaging and multimodal treatment	toxicity of Bi-based NPs not thoroughly evaluated	
Chemo-photothermal therapy	PTX and Indocyanine green	TPGS/HA functionalized PLGA NPs	MNs fabricated with PVP-PVA and PVP K90	Centrifugation method	MDA-MB-231 cells	4T1 tumor-bearing mice	The DMNs containing the NPs achieved enhanced mitochondrial apoptosis and ROS-mediated cytotoxicity	Combined chemotherapy and PTT increased ROS and apoptosis-related protein activity, and it demonstrated strong anticancer efficacy with safe local delivery	No pharmacokinetic and biodistribution data provided, and mechanistic insights into immune response not explored	92

BC – Breast cancer; **DMN** – Dissolvable microneedle; **NP** – Nanoparticle; **PVA** - Polyvinyl alcohol; **PVP** – Polyvinyl pyrrolidone; **HPMC** – Hydroxypropyl methylcellulose; **GelMA** – Gelatin methacrylate; **HA** – Hyaluronic acid; **PNP** – Polymeric nanoparticles; **MCS NPs** – Mannosylated chitosan NPs ; **NIR** – Near infrared; **CT** – Computed tomography; GSH – Glutathione; **SDT** – Sonodynamic therapy; **DC** – Dendritic cell; **PTT** – Photothermal therapy; **PDT** – Photodynamic therapy; **PTX** – Paclitaxel; **ROS** – Reactive oxygen species; **TPGS** – d-alpha-tocopheryl polyethylene glycol succinate

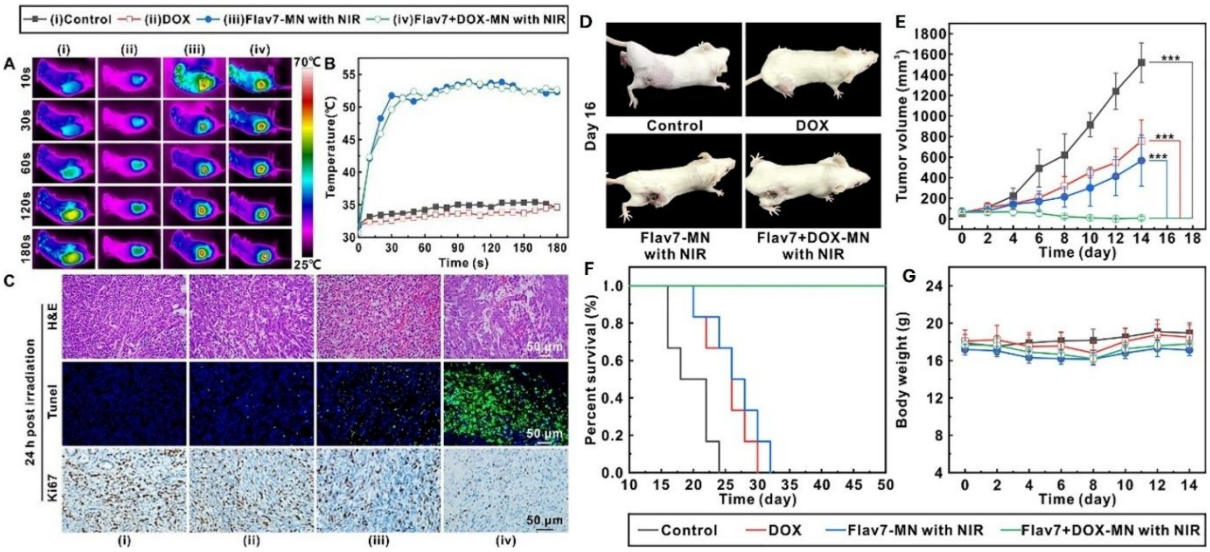

Figure S5. Light-triggered chemo-thermal therapy of breast cancer using MNs. (A) Infrared thermal images of 4T1 tumor-bearing mice following MN treatment under light activation. (B) Time-temperature profiles of tumor sites during irradiation. (C) H&E, TUNEL and Ki67 staining images of the tumor slices. (D) Photographs of the mice on day 16 post-treatments. (E) Time-dependent tumor-volume curves of the mice. (F) Survival curves of the mice (n = 6). (G) Time-dependent body-weight curves of the mice. Adapted with permission from [93]. Copyright 2021, Huixin Wang.

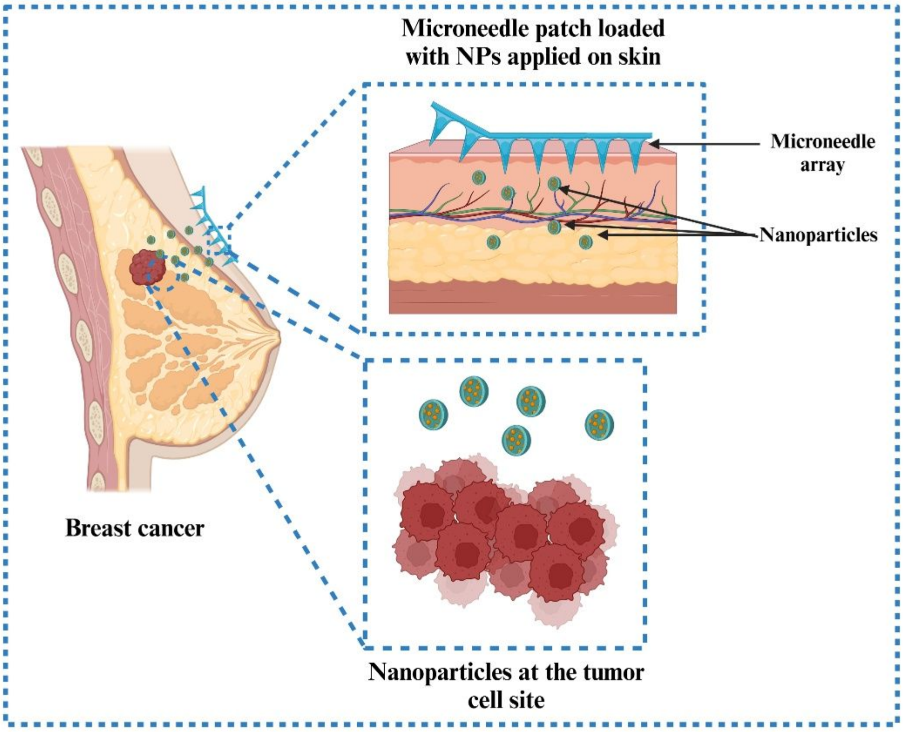

Figure S6. Schematic illustration of MN patch-mediated delivery of NPs for treating BC. The MN patch is placed against the breast skin, thereby penetrating the epidermis and reaching directly beneath the surface into the tissue. Upon application, NPs will accumulate at the tumor site and release therapeutic agents to target the cancer cells. Created using BioRender.

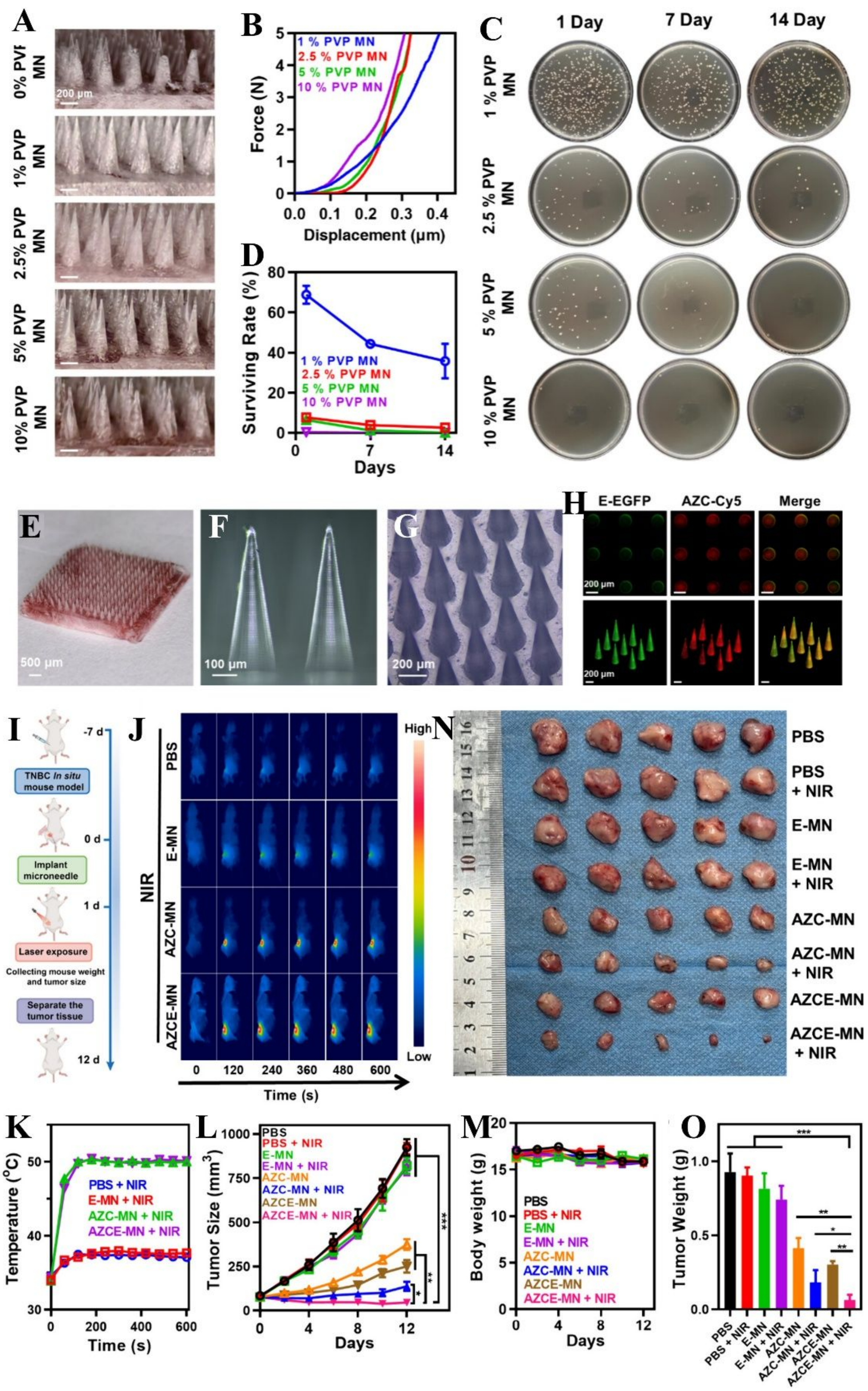

Figure S7. (A) Representative images of MNs, scale bar: 200 μm. (B) Colony formation assay images of surviving *E. coli* for each MN formulation. (C) Quantification of E. coli survival rate across MN groups. (D) Force–displacement profile of MNs. (E) Photographic images of

AZCE-MN, scale bar: 500 µm. (F, G) Array morphology of AZCE-MN, scale bar: 100 and 200 µm. (H) CLSM images of AZCE-MNCy5EGFP. (I) Schematic diagram of the animal experiments setup. (J) Infrared thermographic images of 4T1 tumor-bearing mice post-NIR laser treatments. (K) Temperature profiles of tumor sites during NIR irradiation. (L) The tumor growth inhibition curves in the 4T1 mouse model. (M) Body weight changes of mice during treatments. (N) Photographs of excised 4T1 tumors post-treatment. (O) Weight of excised 4T1 tumors after treatment. The data are shown as the mean ± SEM (n = 5), *P < 0.05, **P < 0.01, ***P < 0.001, and ns indicates no significance. Adapted with permission from [94]. Copyright 2025, American Chemical Society

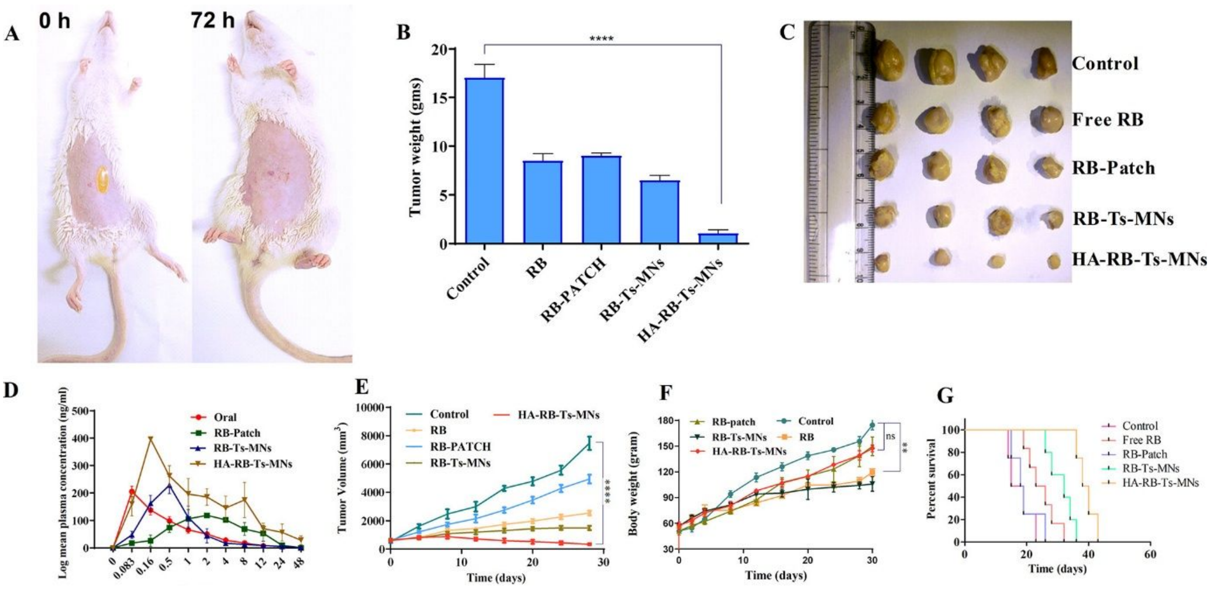

Figure S8. (A) Photography image of an SD rat at 0 and 72 h post-insertion, (B) Tumor weights, (C) Tumor morphology at the study end, and (D) Pharmacokinetic parameter of free RB, RB patch, RB-Ts-MNs, and HA-RB-Ts-MNs in serum at various intervals (50 mg/kg RB equivalent, n=5). *In vivo* antitumor evaluation in female SD rats with LA-7 cell-induced mammary tumor (RB dose: 50 mg/kg). (E) Tumor volume over time, (F) Body weight changes during the study period, and (G) Survival rates of treated LA-7 tumor-bearing SD rats compared to control (n = 4). Adapted with permission from [95]. Copyright 2022, American Chemical Society

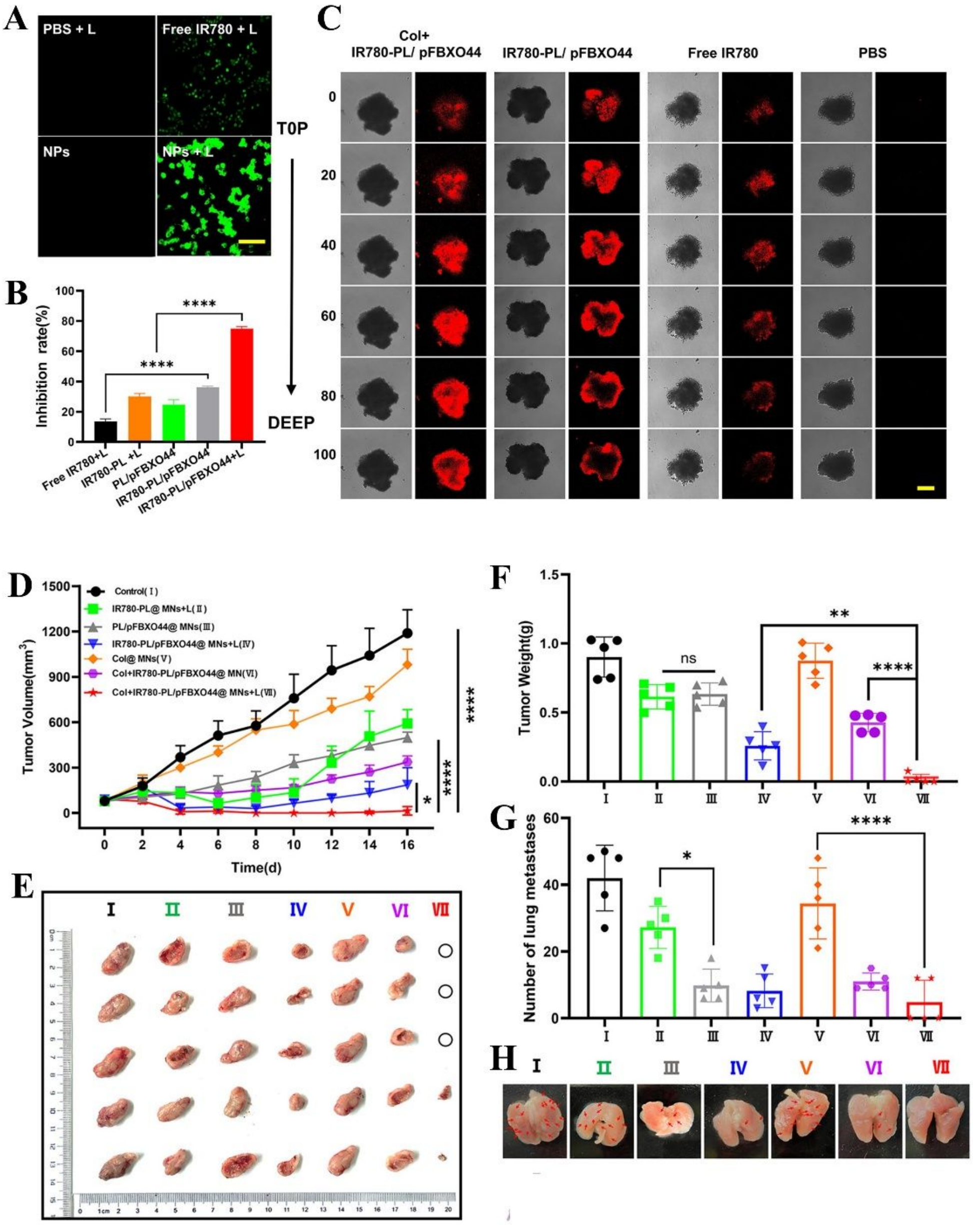

Figure S9. (A) ROS detection in 4T1 cells observed by CLSM. Scale bar: 100 μm. (B) Inhibition rate of 4T1 cells post-treatment with various formulations. (C) Photographs of 4T1 tumor spheroids after incubation of free IR780, IR780-PL/pFBXO44 or Col+IR780-PL/pFBXO44 for 3 h. Scale bar: 200 μm. Data were expressed as mean ± SD, n = 3, $*p < 0.05$, $****p < 0.0001$. (D) Averaged tumor volume curves of mice treated with various MNs (n = 5, $*p < 0.05$, $****p < 0.0001$). (E) Excised tumor images after different treatments. (F) Tumor weight of mice after treatment (n = 5, ns means not significant, $**p < 0.01$, $****p < 0.0001$). (G) Number of lung metastasis nodules (n = 5, $*p < 0.05$, $****p < 0.0001$). (H) Images of the lung in 4T1-tumor-bearing mice post-treatment, metastasis sites marked by red arrows. Adapted with permission from [96]. Copyright 2023, American Chemical Society

References for the Supporting information

(1) Yetisgin, A. A.; Cetinel, S.; Zuvin, M.; Kosar, A.; Kutlu, O. Therapeutic Nanoparticles and Their Targeted Delivery Applications. *Molecules* **2020**, *25* (9), 2193. https://doi.org/10.3390/molecules25092193.

(2) Hsu, C.-Y.; Rheima, A. M.; Kadhim, M. M.; Ahmed, N. N.; Mohammed, S. H.; Abbas, F. H.; Abed, Z. T.; Mahdi, Z. M.; Abbas, Z. S.; Hachim, S. K.; Ali, F. K.; Mahmoud, Z. H.; Kianfar, E. An Overview of Nanoparticles in Drug Delivery: Properties and Applications. *South Afr. J. Chem. Eng.* **2023**, *46*, 233–270. https://doi.org/10.1016/j.sajce.2023.08.009.

(3) Datta, D.; Colaco, V.; Alizadeh, B.; Bandi, S. P.; Dhas, N. 14 - Micro/Nanoparticles. In *Polymers for Oral Drug Delivery Technologies*; Parambath, A., Ed.; Woodhead Publishing Series in Biomaterials; Elsevier Science Ltd, 2025; pp 573–629. https://doi.org/10.1016/B978-0-443-13774-7.00014-1.

(4) Xiao, X.; Teng, F.; Shi, C.; Chen, J.; Wu, S.; Wang, B.; Meng, X.; Essiet Imeh, A.; Li, W. Polymeric Nanoparticles—Promising Carriers for Cancer Therapy. *Front. Bioeng. Biotechnol.* **2022**, *10*. https://doi.org/10.3389/fbioe.2022.1024143.

(5) Colaco, V.; Roy, A. A.; Naik, G. A. R. R.; Mondal, A.; Mutalik, S.; Dhas, N. Advancement in Lipid-Based Nanocomposites for Theranostic Applications in Lung Carcinoma Treatment. *OpenNano* **2024**, *15*, 100199. https://doi.org/10.1016/j.onano.2023.100199.

(6) Mo, K.; Kim, A.; Choe, S.; Shin, M.; Yoon, H. Overview of Solid Lipid Nanoparticles in Breast Cancer Therapy. *Pharmaceutics* **2023**, *15* (8), 2065. https://doi.org/10.3390/pharmaceutics15082065.

(7) Joun, I.; Nixdorf, S.; Deng, W. Advances in Lipid-Based Nanocarriers for Breast Cancer Metastasis Treatment. *Front. Med. Technol.* **2022**, *4*. https://doi.org/10.3389/fmedt.2022.893056.

(8) Zhang, Z.; Yao, S.; Hu, Y.; Zhao, X.; Lee, R. J. Application of Lipid-Based Nanoparticles in Cancer Immunotherapy. *Front. Immunol.* **2022**, *13*, 967505. https://doi.org/10.3389/fimmu.2022.967505.

(9) Nsairat, H.; Khater, D.; Sayed, U.; Odeh, F.; Al Bawab, A.; Alshaer, W. Liposomes: Structure, Composition, Types, and Clinical Applications. *Heliyon* **2022**, *8* (5), e09394. https://doi.org/10.1016/j.heliyon.2022.e09394.

(10) Large, D. E.; Abdelmessih, R. G.; Fink, E. A.; Auguste, D. T. Liposome Composition in Drug Delivery Design, Synthesis, Characterization, and Clinical Application. *Adv. Drug Deliv. Rev.* **2021**, *176*, 113851. https://doi.org/10.1016/j.addr.2021.113851.

(11) Nguyen, T.-T.-L.; Duong, V.-A. Solid Lipid Nanoparticles. *Encyclopedia* **2022**, *2* (2), 952–973. https://doi.org/10.3390/encyclopedia2020063.

(12) Scioli Montoto, S.; Muraca, G.; Ruiz, M. E. Solid Lipid Nanoparticles for Drug Delivery: Pharmacological and Biopharmaceutical Aspects. *Front. Mol. Biosci.* **2020**, *7*. https://doi.org/10.3389/fmolb.2020.587997.

(13) Duan, Y.; Dhar, A.; Patel, C.; Khimani, M.; Neogi, S.; Sharma, P.; Siva Kumar, N.; Vekariya, R. L. A Brief Review on Solid Lipid Nanoparticles: Part and Parcel of Contemporary Drug Delivery Systems. *RSC Adv.* **2020**, *10* (45), 26777–26791. https://doi.org/10.1039/D0RA03491F.

(14) Garg, J.; Pathania, K.; Sah, S. P.; Pawar, S. V. Nanostructured Lipid Carriers: A Promising Drug Carrier for Targeting Brain Tumours. *Future J. Pharm. Sci.* **2022**, *8* (1), 25. https://doi.org/10.1186/s43094-022-00414-8.

(15) Mahor, A. K.; Singh, P. P.; Gupta, R.; Bhardwaj, P.; Rathore, P.; Kishore, A.; Goyal, R.; Sharma, N.; Verma, J.; Rosenholm, J. M.; Bansal, K. K. Nanostructured Lipid Carriers

for Improved Delivery of Therapeutics via the Oral Route. *J. Nanotechnol.* **2023**, *2023* (1), 4687959. https://doi.org/10.1155/2023/4687959.
(16) Chauhan, I.; Yasir, M.; Verma, M.; Singh, A. P. Nanostructured Lipid Carriers: A Groundbreaking Approach for Transdermal Drug Delivery. *Adv. Pharm. Bull.* **2020**, *10* (2), 150–165. https://doi.org/10.34172/apb.2020.021.
(17) Haider, M.; Abdin, S. M.; Kamal, L.; Orive, G. Nanostructured Lipid Carriers for Delivery of Chemotherapeutics: A Review. *Pharmaceutics* **2020**, *12* (3), 288. https://doi.org/10.3390/pharmaceutics12030288.
(18) Beach, M. A.; Nayanathara, U.; Gao, Y.; Zhang, C.; Xiong, Y.; Wang, Y.; Such, G. K. Polymeric Nanoparticles for Drug Delivery. *Chem. Rev.* **2024**, *124* (9), 5505–5616. https://doi.org/10.1021/acs.chemrev.3c00705.
(19) Salari, N.; Faraji, F.; Torghabeh, F. M.; Faraji, F.; Mansouri, K.; Abam, F.; Shohaimi, S.; Akbari, H.; Mohammadi, M. Polymer-Based Drug Delivery Systems for Anticancer Drugs: A Systematic Review. *Cancer Treat. Res. Commun.* **2022**, *32*, 100605. https://doi.org/10.1016/j.ctarc.2022.100605.
(20) Bazzazan, M. A.; Fathollazadeh, P.; Keshavarz Shahbaz, S.; Rezaei, N. Polymeric Nanoparticles as a Promising Platform for Treating Triple-Negative Breast Cancer: Current Status and Future Perspectives. *Int. J. Pharm.* **2024**, *664*, 124639. https://doi.org/10.1016/j.ijpharm.2024.124639.
(21) Alamdari, S. G.; Amini, M.; Jalilzadeh, N.; Baradaran, B.; Mohammadzadeh, R.; Mokhtarzadeh, A.; Oroojalian, F. Recent Advances in Nanoparticle-Based Photothermal Therapy for Breast Cancer. *J. Controlled Release* **2022**, *349*, 269–303. https://doi.org/10.1016/j.jconrel.2022.06.050.
(22) Riley, R. S.; Day, E. S. Gold Nanoparticle-Mediated Photothermal Therapy: Applications and Opportunities for Multimodal Cancer Treatment. *Wiley Interdiscip. Rev. Nanomed. Nanobiotechnol.* **2017**, *9* (4). https://doi.org/10.1002/wnan.1449.
(23) Bustanji, Y.; Taneera, J.; Semreen, M. H.; Abu-Gharbieh, E.; El-Huneidi, W.; Faris, M. A.-I. E.; Alzoubi, K. H.; Soares, N. C.; Albustanji, B.; Abuhelwa, A. Y.; Abu-Zurayk, R.; Alqudah, M. A. Y.; AlKhatib, H. S. Gold Nanoparticles and Breast Cancer: A Bibliometric Analysis of the Current State of Research and Future Directions. *OpenNano* **2023**, *12*, 100164. https://doi.org/10.1016/j.onano.2023.100164.
(24) Narayan, R.; Nayak, U. Y.; Raichur, A. M.; Garg, S. Mesoporous Silica Nanoparticles: A Comprehensive Review on Synthesis and Recent Advances. *Pharmaceutics* **2018**, *10* (3), 118. https://doi.org/10.3390/pharmaceutics10030118.
(25) Manzano, M.; Vallet-Regí, M. Mesoporous Silica Nanoparticles for Drug Delivery. *Adv. Funct. Mater.* **2020**, *30* (2), 1902634. https://doi.org/10.1002/adfm.201902634.
(26) Holmannova, D.; Borsky, P.; Svadlakova, T.; Borska, L.; Fiala, Z. Carbon Nanoparticles and Their Biomedical Applications. *Appl. Sci.* **2022**, *12* (15), 7865. https://doi.org/10.3390/app12157865.
(27) Ayanda, O. S.; Mmuoegbulam, A. O.; Okezie, O.; Durumin Iya, N. I.; Mohammed, S. E.; James, P. H.; Muhammad, A. B.; Unimke, A. A.; Alim, S. A.; Yahaya, S. M.; Ojo, A.; Adaramoye, T. O.; Ekundayo, S. K.; Abdullahi, A.; Badamasi, H. Recent Progress in Carbon-Based Nanomaterials: Critical Review. *J. Nanoparticle Res.* **2024**, *26* (5), 106. https://doi.org/10.1007/s11051-024-06006-2.
(28) Maiti, D.; Tong, X.; Mou, X.; Yang, K. Carbon-Based Nanomaterials for Biomedical Applications: A Recent Study. *Front. Pharmacol.* **2019**, *9*. https://doi.org/10.3389/fphar.2018.01401.
(29) Yang, F.; He, Q.; Dai, X.; Zhang, X.; Song, D. The Potential Role of Nanomedicine in the Treatment of Breast Cancer to Overcome the Obstacles of Current Therapies. *Front. Pharmacol.* **2023**, *14*. https://doi.org/10.3389/fphar.2023.1143102.

(30) Oehler, J. B.; Rajapaksha, W.; Albrecht, H. Emerging Applications of Nanoparticles in the Diagnosis and Treatment of Breast Cancer. *J. Pers. Med.* **2024**, *14* (7), 723. https://doi.org/10.3390/jpm14070723.
(31) Jiang, Y.; Jiang, Z.; Wang, M.; Ma, L. Current Understandings and Clinical Translation of Nanomedicines for Breast Cancer Therapy. *Adv. Drug Deliv. Rev.* **2022**, *180*, 114034. https://doi.org/10.1016/j.addr.2021.114034.
(32) Subhan, M. A.; Yalamarty, S. S. K.; Filipczak, N.; Parveen, F.; Torchilin, V. P. Recent Advances in Tumor Targeting via EPR Effect for Cancer Treatment. *J. Pers. Med.* **2021**, *11* (6), 571. https://doi.org/10.3390/jpm11060571.
(33) Nagpal, D.; Verma, R.; Mittal, V.; Jeandet, P.; Kaushik, D. Targeted Therapies against Breast Cancer: Clinical Perspectives, Obstacles and New Opportunities. *J. Drug Deliv. Sci. Technol.* **2023**, *89*, 105049. https://doi.org/10.1016/j.jddst.2023.105049.
(34) Wei, G.; Wang, Y.; Yang, G.; Wang, Y.; Ju, R. Recent Progress in Nanomedicine for Enhanced Cancer Chemotherapy. *Theranostics* **2021**, *11* (13), 6370–6392. https://doi.org/10.7150/thno.57828.
(35) Shi, J.; Kantoff, P. W.; Wooster, R.; Farokhzad, O. C. Cancer Nanomedicine: Progress, Challenges and Opportunities. *Nat. Rev. Cancer* **2017**, *17* (1), 20–37. https://doi.org/10.1038/nrc.2016.108.
(36) Gavas, S.; Quazi, S.; Karpiński, T. M. Nanoparticles for Cancer Therapy: Current Progress and Challenges. *Nanoscale Res. Lett.* **2021**, *16* (1), 173. https://doi.org/10.1186/s11671-021-03628-6.
(37) Jia, J.; Wang, Z.; Yue, T.; Su, G.; Teng, C.; Yan, B. Crossing Biological Barriers by Engineered Nanoparticles. *Chem. Res. Toxicol.* **2020**, *33* (5), 1055–1060. https://doi.org/10.1021/acs.chemrestox.9b00483.
(38) Alimardani, V.; Abolmaali, S. S.; Yousefi, G.; Rahiminezhad, Z.; Abedi, M.; Tamaddon, A.; Ahadian, S. Microneedle Arrays Combined with Nanomedicine Approaches for Transdermal Delivery of Therapeutics. *J. Clin. Med.* **2021**, *10* (2), 181. https://doi.org/10.3390/jcm10020181.
(39) Jung, J. H.; Jin, S. G. Microneedle for Transdermal Drug Delivery: Current Trends and Fabrication. *J. Pharm. Investig.* **2021**, *51* (5), 503–517. https://doi.org/10.1007/s40005-021-00512-4.
(40) Prausnitz, M. R. Engineering Microneedle Patches for Vaccination and Drug Delivery to Skin. *Annu. Rev. Chem. Biomol. Eng.* **2017**, *8* (Volume 8, 2017), 177–200. https://doi.org/10.1146/annurev-chembioeng-060816-101514.
(41) Hoang, M. T.; Ita, K. B.; Bair, D. A. Solid Microneedles for Transdermal Delivery of Amantadine Hydrochloride and Pramipexole Dihydrochloride. *Pharmaceutics* **2015**, *7* (4), 379–396. https://doi.org/10.3390/pharmaceutics7040379.
(42) Bilal, M.; Mehmood, S.; Raza, A.; Hayat, U.; Rasheed, T.; Iqbal, H. M. N. Microneedles in Smart Drug Delivery. *Adv. Wound Care* **2021**, *10* (4), 204–219. https://doi.org/10.1089/wound.2019.1122.
(43) Meyer, B. K.; Kendall, M. A. F.; Williams, D. M.; Bett, A. J.; Dubey, S.; Gentzel, R. C.; Casimiro, D.; Forster, A.; Corbett, H.; Crichton, M.; Baker, S. B.; Evans, R. K.; Bhambhani, A. Immune Response and Reactogenicity of an Unadjuvanted Intradermally Delivered Human Papillomavirus Vaccine Using a First Generation Nanopatch™ in Rhesus Macaques: An Exploratory, Pre-Clinical Feasibility Assessment. *Vaccine X* **2019**, *2*, 100030. https://doi.org/10.1016/j.jvacx.2019.100030.
(44) Caudill, C. L.; Perry, J. L.; Tian, S.; Luft, J. C.; DeSimone, J. M. Spatially Controlled Coating of Continuous Liquid Interface Production Microneedles for Transdermal Protein Delivery. *J. Controlled Release* **2018**, *284*, 122–132. https://doi.org/10.1016/j.jconrel.2018.05.042.

(45) Kapoor, Y.; Milewski, M.; Dick, L.; Zhang, J.; Bothe, J. R.; Gehrt, M.; Manser, K.; Nissley, B.; Petrescu, I.; Johnson, P.; Burton, S.; Moseman, J.; Hua, V.; Grunewald, T.; Tomai, M.; Smith, R. Coated Microneedles for Transdermal Delivery of a Potent Pharmaceutical Peptide. *Biomed. Microdevices* **2019**, *22* (1), 7. https://doi.org/10.1007/s10544-019-0462-1.
(46) Mönkäre, J.; Pontier, M.; van Kampen, E. E. M.; Du, G.; Leone, M.; Romeijn, S.; Nejadnik, M. R.; O'Mahony, C.; Slütter, B.; Jiskoot, W.; Bouwstra, J. A. Development of PLGA Nanoparticle Loaded Dissolving Microneedles and Comparison with Hollow Microneedles in Intradermal Vaccine Delivery. *Eur. J. Pharm. Biopharm.* **2018**, *129*, 111–121. https://doi.org/10.1016/j.ejpb.2018.05.031.
(47) Du, G.; Woythe, L.; van der Maaden, K.; Leone, M.; Romeijn, S.; Kros, A.; Kersten, G.; Jiskoot, W.; Bouwstra, J. A. Coated and Hollow Microneedle-Mediated Intradermal Immunization in Mice with Diphtheria Toxoid Loaded Mesoporous Silica Nanoparticles. *Pharm. Res.* **2018**, *35* (10), 189. https://doi.org/10.1007/s11095-018-2476-4.
(48) Liu, T.; Chen, M.; Fu, J.; Sun, Y.; Lu, C.; Quan, G.; Pan, X.; Wu, C. Recent Advances in Microneedles-Mediated Transdermal Delivery of Protein and Peptide Drugs. *Acta Pharm. Sin. B* **2021**, *11* (8), 2326–2343. https://doi.org/10.1016/j.apsb.2021.03.003.
(49) Oliveira, C.; Teixeira, J. A.; Oliveira, N.; Ferreira, S.; Botelho, C. M. Microneedles' Device: Design, Fabrication, and Applications. *Macromol* **2024**, *4* (2), 320–355. https://doi.org/10.3390/macromol4020019.
(50) Wang, Q. L.; Zhu, D. D.; Liu, X. B.; Chen, B. Z.; Guo, X. D. Microneedles with Controlled Bubble Sizes and Drug Distributions for Efficient Transdermal Drug Delivery. *Sci. Rep.* **2016**, *6* (1), 38755. https://doi.org/10.1038/srep38755.
(51) He, R.; Niu, Y.; Li, Z.; Li, A.; Yang, H.; Xu, F.; Li, F. A Hydrogel Microneedle Patch for Point-of-Care Testing Based on Skin Interstitial Fluid. *Adv. Healthc. Mater.* **2020**, *9* (4), 1901201. https://doi.org/10.1002/adhm.201901201.
(52) Oliveira, C.; Teixeira, J. A.; Oliveira, N.; Ferreira, S.; Botelho, C. M. Microneedles' Device: Design, Fabrication, and Applications. *Macromol* **2024**, *4* (2), 320–355. https://doi.org/10.3390/macromol4020019.
(53) Avcil, M.; Çelik, A. Microneedles in Drug Delivery: Progress and Challenges. *Micromachines* **2021**, *12* (11), 1321. https://doi.org/10.3390/mi12111321.
(54) Waghule, T.; Singhvi, G.; Dubey, S. K.; Pandey, M. M.; Gupta, G.; Singh, M.; Dua, K. Microneedles: A Smart Approach and Increasing Potential for Transdermal Drug Delivery System. *Biomed. Pharmacother.* **2019**, *109*, 1249–1258. https://doi.org/10.1016/j.biopha.2018.10.078.
(55) Li, J.; Zeng, M.; Shan, H.; Tong, C. Microneedle Patches as Drug and Vaccine Delivery Platform. *Curr. Med. Chem.* **2017**, *24* (22). https://doi.org/10.2174/0929867324666170526124053.
(56) Pradeep Narayanan, S.; Raghavan, S. Solid Silicon Microneedles for Drug Delivery Applications. *Int. J. Adv. Manuf. Technol.* **2017**, *93* (1), 407–422. https://doi.org/10.1007/s00170-016-9698-6.
(57) Tucak, A.; Sirbubalo, M.; Hindija, L.; Rahić, O.; Hadžiabdić, J.; Muhamedagić, K.; Čekić, A.; Vranić, E. Microneedles: Characteristics, Materials, Production Methods and Commercial Development. *Micromachines* **2020**, *11* (11), 961. https://doi.org/10.3390/mi11110961.
(58) Cárcamo-Martínez, Á.; Mallon, B.; Domínguez-Robles, J.; Vora, L. K.; Anjani, Q. K.; Donnelly, R. F. Hollow Microneedles: A Perspective in Biomedical Applications. *Int. J. Pharm.* **2021**, *599*, 120455. https://doi.org/10.1016/j.ijpharm.2021.120455.
(59) Iachina, I.; Eriksson, A. H.; Bertelsen, M.; Petersson, K.; Jansson, J.; Kemp, P.; Engell, K. M.; Brewer, J. R.; Nielsen, K. T. Dissolvable Microneedles for Transdermal Drug

Delivery Showing Skin Penetration and Modified Drug Release. *Eur. J. Pharm. Sci.* **2023**, *182*, 106371. https://doi.org/10.1016/j.ejps.2023.106371.

(60) Dave, R.; Shinde, S.; Kalayil, N.; Budar, A. Engineering Microscopic Delivery Systems: A Review of Dissolving Microneedle Design, Fabrication, and Function. *Micro Nano Syst. Lett.* **2024**, *12* (1), 14. https://doi.org/10.1186/s40486-024-00204-2.

(61) Mohite, P.; Puri, A.; Munde, S.; Ade, N.; Kumar, A.; Jantrawut, P.; Singh, S.; Chittasupho, C. Hydrogel-Forming Microneedles in the Management of Dermal Disorders Through a Non-Invasive Process: A Review. *Gels* **2024**, *10* (11), 719. https://doi.org/10.3390/gels10110719.

(62) Loh, J. M.; Lim, Y. J. L.; Tay, J. T.; Cheng, H. M.; Tey, H. L.; Liang, K. Design and Fabrication of Customizable Microneedles Enabled by 3D Printing for Biomedical Applications. *Bioact. Mater.* **2024**, *32*, 222–241. https://doi.org/10.1016/j.bioactmat.2023.09.022.

(63) Bauleth-Ramos, T.; El-Sayed, N.; Fontana, F.; Lobita, M.; Shahbazi, M.-A.; Santos, H. A. Recent Approaches for Enhancing the Performance of Dissolving Microneedles in Drug Delivery Applications. *Mater. Today* **2023**, *63*, 239–287. https://doi.org/10.1016/j.mattod.2022.12.007.

(64) Loizidou, E. Z.; Inoue, N. T.; Ashton-Barnett, J.; Barrow, D. A.; Allender, C. J. Evaluation of Geometrical Effects of Microneedles on Skin Penetration by CT Scan and Finite Element Analysis. *Eur. J. Pharm. Biopharm.* **2016**, *107*, 1–6. https://doi.org/10.1016/j.ejpb.2016.06.023.

(65) Tamez-Tamez, J. I.; Vázquez-Lepe, E.; Rodriguez, C. A.; Martínez-López, J. I.; García-López, E. Assessment of Geometrical Dimensions and Puncture Feasibility of Microneedles Manufactured by Micromilling. *Int. J. Adv. Manuf. Technol.* **2023**, *126* (11), 4983–4996. https://doi.org/10.1007/s00170-023-11467-1.

(66) Li, Y.; Hu, X.; Dong, Z.; Chen, Y.; Zhao, W.; Wang, Y.; Zhang, L.; Chen, M.; Wu, C.; Wang, Q. Dissolving Microneedle Arrays with Optimized Needle Geometry for Transcutaneous Immunization. *Eur. J. Pharm. Sci.* **2020**, *151*, 105361. https://doi.org/10.1016/j.ejps.2020.105361.

(67) Potts, M. R.; Evans, S. L.; Pullin, R.; Coulman, S. A.; Birchall, J. C.; Wyatt, H. An Analysis of the Relationship between Microneedle Spacing, Needle Force and Skin Strain during the Indentation Phase Prior to Skin Penetration. *Comput. Methods Biomech. Biomed. Engin.* **2023**, *26* (14), 1719–1731. https://doi.org/10.1080/10255842.2022.2136486.

(68) Verbaan, F. J.; Bal, S. M.; van den Berg, D. J.; Groenink, W. H. H.; Verpoorten, H.; Lüttge, R.; Bouwstra, J. A. Assembled Microneedle Arrays Enhance the Transport of Compounds Varying over a Large Range of Molecular Weight across Human Dermatomed Skin. *J. Controlled Release* **2007**, *117* (2), 238–245. https://doi.org/10.1016/j.jconrel.2006.11.009.

(69) Shu, W.; Heimark, H.; Bertollo, N.; Tobin, D. J.; O'Cearbhaill, E. D.; Annaidh, A. N. Insights into the Mechanics of Solid Conical Microneedle Array Insertion into Skin Using the Finite Element Method. *Acta Biomater.* **2021**, *135*, 403–413. https://doi.org/10.1016/j.actbio.2021.08.045.

(70) Choo, S.; Jin, S.; Jung, J. Fabricating High-Resolution and High-Dimensional Microneedle Mold through the Resolution Improvement of Stereolithography 3D Printing. *Pharmaceutics* **2022**, *14* (4), 766. https://doi.org/10.3390/pharmaceutics14040766.

(71) Choo, S.; Jin, S.; Jung, J. Fabricating High-Resolution and High-Dimensional Microneedle Mold through the Resolution Improvement of Stereolithography 3D

Printing. *Pharmaceutics* **2022**, *14* (4), 766. https://doi.org/10.3390/pharmaceutics14040766.
(72) Bhatnagar, S.; Bankar, N. G.; Kulkarni, M. V.; Venuganti, V. V. K. Dissolvable Microneedle Patch Containing Doxorubicin and Docetaxel Is Effective in 4T1 Xenografted Breast Cancer Mouse Model. *Int. J. Pharm.* **2019**, *556*, 263–275. https://doi.org/10.1016/j.ijpharm.2018.12.022.
(73) Luo, X.; Yang, L.; Cui, Y. Microneedles: Materials, Fabrication, and Biomedical Applications. *Biomed. Microdevices* **2023**, *25* (3), 20. https://doi.org/10.1007/s10544-023-00658-y.
(74) Nagarkar, R.; Singh, M.; Nguyen, H. X.; Jonnalagadda, S. A Review of Recent Advances in Microneedle Technology for Transdermal Drug Delivery. *J. Drug Deliv. Sci. Technol.* **2020**, *59*, 101923. https://doi.org/10.1016/j.jddst.2020.101923.
(75) Tarbox, T. N.; Watts, A. B.; Cui, Z.; Williams, R. O. An Update on Coating/Manufacturing Techniques of Microneedles. *Drug Deliv. Transl. Res.* **2018**, *8* (6), 1828–1843. https://doi.org/10.1007/s13346-017-0466-4.
(76) Wu, L.; Takama, N.; Park, J.; Kim, B.; Kim, J.; Jeong, D. Shadow Mask Assisted Droplet-Born Air-Blowing Method for Fabrication of Dissoluble Microneedle. *2017 IEEE 12th Int. Conf. NanoMicro Eng. Mol. Syst. NEMS* **2017**, 456–459. https://doi.org/10.1109/NEMS.2017.8017064.
(77) Johnson, A. R.; Caudill, C. L.; Tumbleston, J. R.; Bloomquist, C. J.; Moga, K. A.; Ermoshkin, A.; Shirvanyants, D.; Mecham, S. J.; Luft, J. C.; DeSimone, J. M. Single-Step Fabrication of Computationally Designed Microneedles by Continuous Liquid Interface Production. *PLOS ONE* **2016**, *11* (9), e0162518. https://doi.org/10.1371/journal.pone.0162518.
(78) Lyu, S.; Dong, Z.; Xu, X.; Bei, H.-P.; Yuen, H.-Y.; James Cheung, C.-W.; Wong, M.-S.; He, Y.; Zhao, X. Going below and beyond the Surface: Microneedle Structure, Materials, Drugs, Fabrication, and Applications for Wound Healing and Tissue Regeneration. *Bioact. Mater.* **2023**, *27*, 303–326. https://doi.org/10.1016/j.bioactmat.2023.04.003.
(79) Ruggiero, F.; Vecchione, R.; Bhowmick, S.; Coppola, G.; Coppola, S.; Esposito, E.; Lettera, V.; Ferraro, P.; Netti, P. A. Electro-Drawn Polymer Microneedle Arrays with Controlled Shape and Dimension. *Sens. Actuators B Chem.* **2018**, *255*, 1553–1560. https://doi.org/10.1016/j.snb.2017.08.165.
(80) Apolinário, A. C.; Naser, Y. A.; Volpe-Zanutto, F.; Vora, L. K.; Sabri, A. H.; Li, M.; Hutton, A. R. J.; McCarthy, H. O.; Lopes, L. B.; Donnelly, R. F. Novel Lipid Nanovesicle-Loaded Dissolving Microarray Patches for Fenretinide in Breast Cancer Chemoprevention. *J. Controlled Release* **2024**, *374*, 76–88. https://doi.org/10.1016/j.jconrel.2024.07.080.
(81) Heikal, L. A.; Ashour, A. A.; Aboushanab, A. R.; El-Kamel, A. H.; Zaki, I. I.; El-Moslemany, R. M. Microneedles Integrated with Atorvastatin-Loaded Pumpkisomes for Breast Cancer Therapy: A Localized Delivery Approach. *J. Controlled Release* **2024**, *376*, 354–368. https://doi.org/10.1016/j.jconrel.2024.10.013.
(82) Altameemi, K. K. A.; Abd-Alhammid, S. N. Anastrozole Nanoparticles for Transdermal Delivery through Microneedles: Preparation and Evaluation. *J. Pharm. Negat. Results* **2022**, *13* (3), 974–980. https://doi.org/10.47750/pnr.2022.13.03.152.
(83) Patil, A.; Prabhakar, B.; Shende, P. Potential of Transpapillary Route for Artesunate-Loaded Microneedles against Breast Cancer Cell Line. *Colloids Surf. Physicochem. Eng. Asp.* **2022**, *640*, 128431. https://doi.org/10.1016/j.colsurfa.2022.128431.
(84) Gadag, S.; Narayan, R.; Nayak, A. S.; Catalina Ardila, D.; Sant, S.; Nayak, Y.; Garg, S.; Nayak, U. Y. Development and Preclinical Evaluation of Microneedle-Assisted Resveratrol Loaded Nanostructured Lipid Carriers for Localized Delivery to Breast

Cancer Therapy. *Int. J. Pharm.* **2021**, *606*, 120877. https://doi.org/10.1016/j.ijpharm.2021.120877.

(85) Wang, J.; Wen, T.; Chen, H.; Huang, S.; Guo, R.; Zheng, Y.; Xiao, Z.; Shuai, X. Microneedles-Mediated Intradermal Delivery of Paclitaxel/Anti-PD-1 for Efficient and Safe Triple-Negative Breast Cancer Therapy. *Adv. Ther.* **2024**, *7* (4), 2300362. https://doi.org/10.1002/adtp.202300362.

(86) Yin, Y.; Tang, L.; Cao, Y.; Liu, H.; Fu, C.; Feng, J.; Zhu, H.; Wang, W. Microneedle Patch-Involved Local Therapy Synergized with Immune Checkpoint Inhibitor for Pre- and Post-Operative Cancer Treatment. *J. Controlled Release* **2025**, *379*, 678–695. https://doi.org/10.1016/j.jconrel.2025.01.051.

(87) Chen, Z.; Hu, F.; Xiang, J.; Zhou, X.; Wu, B.; Fan, B.; Tang, H.; Liu, B.; Chen, L. Mesoporous Microneedles Enabled Localized Controllable Delivery of Stimulator of Interferon Gene Agonist Nanoexosomes for FLASH Radioimmunotherapy against Breast Cancer. *ACS Appl. Mater. Interfaces* **2024**, *16* (43), 58180–58190. https://doi.org/10.1021/acsami.4c09833.

(88) Weng, J.; Yang, J.; Wang, W.; Wen, J.; Fang, M.; Zheng, G.; Xie, J.; Zheng, X.; Feng, L.; Yan, Q. Application of Microneedles Combined with Dendritic Cell-Targeted Nanovaccine Delivery System in Percutaneous Immunotherapy for Triple-Negative Breast Cancer. *Nanotechnology* **2023**, *34* (47), 475101. https://doi.org/10.1088/1361-6528/ace97b.

(89) Chu, Z.; Zheng, W.; Fu, W.; Liang, J.; Wang, W.; Xu, L.; Jiang, X.; Zha, Z.; Qian, H. Implanted Microneedles Loaded with Sparfloxacin and Zinc-Manganese Sulfide Nanoparticles Activates Immunity for Postoperative Triple-Negative Breast Cancer to Prevent Recurrence and Metastasis. *Adv. Sci.* **2025**, *12* (16), 2416270. https://doi.org/10.1002/advs.202416270.

(90) Chen, S.-X.; Ma, M.; Xue, F.; Shen, S.; Chen, Q.; Kuang, Y.; Liang, K.; Wang, X.; Chen, H. Construction of Microneedle-Assisted Co-Delivery Platform and Its Combining Photodynamic/Immunotherapy. *J. Controlled Release* **2020**, *324*, 218–227. https://doi.org/10.1016/j.jconrel.2020.05.006.

(91) Hu, T.; Jia, L.; Li, H.; Yang, C.; Yan, Y.; Lin, H.; Zhang, F.; Qu, F.; Guo, W. An Intelligent and Soluble Microneedle Composed of Bi/BiVO4 Schottky Heterojunction for Tumor Ct Imaging and Starvation/Gas Therapy-Promoted Synergistic Cancer Treatment. *Adv. Healthc. Mater.* **2024**, *13* (8), 2303147. https://doi.org/10.1002/adhm.202303147.

(92) Peng, T.; Huang, Y.; Feng, X.; Zhu, C.; Yin, S.; Wang, X.; Bai, X.; Pan, X.; Wu, C. TPGS/Hyaluronic Acid Dual-Functionalized PLGA Nanoparticles Delivered through Dissolving Microneedles for Markedly Improved Chemo-Photothermal Combined Therapy of Superficial Tumor. *Acta Pharm. Sin. B* **2021**, *11* (10), 3297–3309. https://doi.org/10.1016/j.apsb.2020.11.013.

(93) Wang, H.; Wang, W.; Li, C.; Xu, A.; Qiu, B.; Li, F.; Ding, W. Flav7 + DOX Co-Loaded Separable Microneedle for Light-Triggered Chemo-Thermal Therapy of Superficial Tumors. *Chem. Eng. J.* **2022**, *428*, 131913. https://doi.org/10.1016/j.cej.2021.131913.

(94) Zhang, W.; Jiang, Y.; Liu, L.; Shen, H.; Huang, X.; Zheng, W.; Chu, Z.; Wang, W.; Guo, Y.; Qian, H. Implantable Microneedles Loaded with Nanoparticles Surface Engineered Escherichia Coli for Efficient Eradication of Triple-Negative Breast Cancer Stem Cells. *Nano Lett.* **2025**, *25* (5), 2041–2051. https://doi.org/10.1021/acs.nanolett.4c06052.

(95) Sharma, M.; Mittapelly, N.; Banala, V. T.; Urandur, S.; Gautam, S.; Marwaha, D.; Rai, N.; Singh, N.; Gupta, A.; Mitra, K.; Mishra, P. R. Amalgamated Microneedle Array Bearing Ribociclib-Loaded Transfersomes Eradicates Breast Cancer via CD44 Targeting. *Biomacromolecules* **2022**, *23* (3), 661–675. https://doi.org/10.1021/acs.biomac.1c01076.

(96) Wang, T.; Chen, G.; Zhang, S.; Li, D.; Wei, G.; Zhao, X.; Liu, Y.; Ding, D.; Zhang, X. Steerable Microneedles Enabling Deep Delivery of Photosensitizers and CRISPR/Cas9 Systems for Effective Combination Cancer Therapy. *Nano Lett.* **2023**, *23* (17), 7990–7999. https://doi.org/10.1021/acs.nanolett.3c01914.
